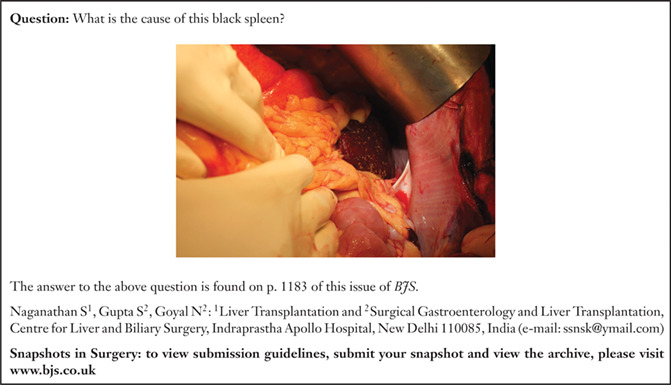# Multicentre prospective cohort study of body mass index and postoperative complications following gastrointestinal surgery

**DOI:** 10.1002/bjs.10203

**Published:** 2016-06-20

**Authors:** STARSurg Collaborative, T M Drake, D Nepogodiev, S J Chapman, J C Glasbey, C Khatri, C Y Kong, H A Claireaux, M F Bath, M Mohan, L McNamee, M Kelly, H Mitchell, J E Fitzgerald, E M Harrison, A Bhangu, H A Claireaux, I Antoniou, R Dean, N Davies, S Trecarten, I Henderson, C Holmes, J Wylie, R H Shuttleworth, A Jindal, F Hughes, P Gouda, L McNamee, R Fleck, M Hanrahan, P Karunakaran, J H Chen, M C Sykes, R K Sethi, S Suresh, P Patel, M Patel, R K Varma, J Mushtaq, B Gundogan, W Bolton, M Mohan, T Khan, J Burke, R Morley, N Favero, R Adams, V Thirumal, E D Kennedy, K K Ong, Y H Tan, J Gabriel, A Bakhsh, J Y L Low, A Yener, V Paraoan, R Preece, T W Tilston, E Cumber, S Dean, T Ross, E McCance, H Amin, L Satterthwaite, K D Clement, R Gratton, E D Mills, S M Chiu, G Hung, N M Rafiq, J D B Hayes, K L Robertson, K Dynes, H C Huang, S Assadullah, J W Duncumb, R D C Moon, S X Poo, J K Mehta, K R Joshi, R Callan, J M Norris, N J Chilvers, H Keevil, P Jull, S Mallick, D Elf, L Carr, C Player, E C Barton, A L Martin, S G Ratu, E J Roberts, P N Phan, A R Dyal, J E Rogers, A D Henson, N B Reid, D Burke, G Culleton, S Lynne, D Burke, S Mansoor, C Brennan, R Blessed, C Holloway, A Hill, T Goldsmith, S Mackin, S Kim, E Woin, G Brent, J Coffin, O Ziff, Z Momoh, R Debenham, M Ahmed, C S Yong, J C Wan, H C Copley, P Raut, F I Chaudhry, R H Shuttleworth, G Nixon, C Dorman, R Tan, S Kanabar, N Canning, M Dolaghan, N Bell, M McMenamin, A Chhabra, K Duke, L Turner, T Patel, L S Chew, M Mirza, S Lunawat, B Oremule, N Ward, M Khan, E T Tan, D Maclennan, R J McGregor, E G Chisholm, E J Griffin, L Bell, B A Hughes, J Davies, H Haq, H Ahmed, N Ungcharoen, C Whacha, R Thethi, R M Markham, A H Y Lee, E Batt, N P Bullock, C T Francescon, J E Davies, N M Shafiq, J Zhao, S Vivekanantham, I Barai, J L Y Allen, D C Marshall, C J McIntyre, H C P Wilson, A J Ashton, C Lek, N Behar, M Davis-Hall, N Seneviratne, S Kim, L Esteve, M Sirakaya, S Ali, S Pope, J S Ahn, A Craig-McQuaide, W A Gatfield, S Leong, A M Demetri, A L Kerr, C Rees, J Loveday, S Liu, M Wijesekera, D Maru, M Attalla, N Smith, D Brown, P Sritharan, A Shah, V Charavanamuttu, G Heppenstall-Harris, K Ng, T Raghvani, N Rajan, K Hulley, N Moody, M Williams, A Cotton, M Sharifpour, K N Lwin, M Bright, A R Chitnis, M Abdelhadi, A D Semana, F Morgan, R Reid, J Dickson, L Anderson, R McMullan, J Dickson, N Ahern, A Asmadi, L B Anderson, J Lua Boon Xuan, L Crozier, S McAleer, D M Lees, A A Adebayo, M Das, A H Amphlett, A Al-Robeye, A Valli, J Khangura, A Winarski, A Ali, J Khangura, H Woodward, C Gouldthrope, M Turner, K Sasapu, M Tonkins, J R L Wild, M Robinson, J Hardie, R Heminway, R Narramore, N Ramjeeawon, A Hibberd, F Winslow, W Ho, B F Chong, K Lim, S Ho, J A Crewdson, S Singagireson, N Kalra, F Koumpa, H Jhala, W C Soon, M Karia, M G Rasiah, D Xylas, H Gilbert, M Sundar-Singh, J Wills, J Mushtaq, S Akhtar, S Patel, L Hu, C Brathwaite-Shirley, H Nayee, O Amin, T Rangan, E J H Turner, C McCrann, R Shepherd, N Patel, J Prest-Smith, E Auyoung, A Murtaza, A Coates, O Prys-Jones, M King, S Gaffney, C J Dewdney, I Nehikhare, J Lavery, J Bassett, K Davies, K Ahmad, A Collins, M Acres, C Egerton, T Khan, K Cheng, X Chen, N Chan, A Sheldon, S Khan, J Empey, E Ingram, A Malik, M Johnstone, R Goodier, J P Shah, J E Giles, J A Sanders, S W McLure, S Pal, A Rangedara, A N Baker, C A Asbjoernsen, C Girling, L Gray, L Gauntlett, C Joyner, S Qureshi, S Dean, Y P Mogan, J C K Ng, A N Kumar, J H Park, D Tan, K P Choo, K P Raman, P Buakuma, C Xiao, S Govinden, O D Thompson, M A Charalambos, E Brown, R B Karsan, T Dogra, L M Bullman, P M Dawson, A L Frank, H Abid, L Tung, U Qureshi, A Tahmina, B W Matthews, R T Harris, A O'Connor, K Mazan, S Iqbal, S A Stanger, J D Thompson, J A L Sullivan, E Uppal, A MacAskill, F A Bamgbose, C Neophytou, A F Carroll, C W Rookes, U Datta, A J Dhutia, S Rashid, N Ahmed, T Lo, S Bhanderi, C D Blore, S Ahmed, H Shaheen, S Abburu, S Majid, Z Abbas, S S Talukdar, S Ahmed, L J Burney, J B Patel, O Al-Obaedi, A W Roberts, O Al-Obaedi, S Mahboob, B Singh, S Sheth, P Karia, A Prabhudesai, K Kow, K Koysombat, S Wang, P Morrison, Y Maheswaran, P Keane, P C Copley, O Brewster, G X Xu, P Harries, C Wall, A Al-Mousawi, S Bonsu, P Cunha, T Ward, J Paul, K Nadanakumaran, S Tayeh, T Ward, H Holyoak, J Remedios, K Theodoropoulou, T Ward, A Luhishi, L Jacob, F Long, A Atayi, S Sarwar, O Parker, J Harvey, H Ross, R Rampal, G Thomas, P Vanmali, C McGowan, J Stein, V Robertson, L Carthew, V Teng, J Fong, A N Street, C E Thakker, D O'Reilly, M Bravo, A Pizzolato, H A Khokhar, M Ryan, L Cheskes, R Carr, A E Salih, S Bassiony, R Yuen, D Chrastek, H Rosen O'Sullivan, A Amajuoyi, A Wang, O Sitta, J Wye, M A Qamar, C Major, A Kaushal, C Morgan, M Petrarca, R Allot, K Verma, S Dutt, R Allot, C P Chilima, S Peroos, R Allot, S R Kosasih, H Chin, L Ashken, R J Pearse, R A O'Loughlin, A Menon, K Singh, J Norton, R Sagar, N Jathanna, L Rothwell, N Watson, F Harding, P Dube, H Khalid, N Punjabi, M Sagmeister, P Gill, S Shahid, S Hudson-Phillips, D George, J Ashwood, T Lewis, M Dhar, P Sangal, I A Rhema, D Kotecha, R Dean, Z Afzal, J A Syeed, E Prakash, P Jalota, R Dean, J Herron, L Kimani, A Delport, A Shukla, V Agarwal, S Parthiban, H Thakur, W Cymes, S Rinkoff, J A Turnbull, M Hayat, S Darr, U Khan, J Lim, A Higgins, G Lakshmipathy, B Forte, E Canning, A Jaitley, J Lamont, E Toner, A Ghaffar, M McDowell, D Salmon, P Gouda, O O'Carroll, A Khan, M E Kelly, K Clesham, C Palmer, R Lyons, M E Kelly, A Bell, R Chin, R M Waldron, M E Kelly, A Trimble, S E Cox, U Ashfaq, J Campbell, R B S Holliday, G McCabe, F Morris, R Priestland, S Dean, O K Vernon, A Ledsam, R Vaughan, D Lim, Z R Bakewell, R K Hughes, R M Koshy, H R Jackson, P Narayan, A E Cardwell, C L Jubainville, T Arif, L E Elliott, V Gupta, T Arif, G Bhaskaran, K Singh, A Odeleye, F Ahmed, R Shah, A Odeleye, J Pickard, Y N Suleman, A Odeleye, A S North, L F McClymont, N Hussain, I Ibrahim, G S Ng, V Wong, A E Lim, L N Harris, T Tharmachandirar, D Mittapalli, V Patel, M Lakhani, N Davies, H Z Bazeer, V Narwani, K K Sandhu, L R Wingfield, S Gentry, H Adjei, M Bhatti, L Braganza, J Barnes, S Mistry, G Chillarge, S Stokes, J Cleere, S Wadanamby, A M Bucko, J Meek, N Boxall, E G Heywood, J J Wiltshire, C Toh, A E Ward, B N Shurovi, T M Drake, D Horth, B Y Patel, B Ali, T Spencer, T Axelson, L Kretzmer, C Chhina, C Anandarajah, T Fautz, C Horst, A A Thevathasan, J Q Ng, F Hirst, C F Brewer, A E Logan, J W Lockey, P R Forrest, N Keelty, A D Wood, L R Springford, P Avery, T M Schulz, T P Bemand, L Howells, H Collier, A Khajuria, R G Tharakan, S Parsons, A M Buchan, R J McGalliard, J D Mason, O J Cundy, N Li, N A Redgrave, R P Watson, T P Pezas, Y F Dennis, E Segall, M Hameed, A S Lynch, M Chamberlain, F S Peck, Y N Neo, G Russell, M Elseedawy, S Lee, N L Foster, Y H Soo, L Puan, R Dennis, H Goradia, A Qureshi, S Osman, T Reeves, L Dinsmore, M Marsden, Q Lu, T Pitts-Tucker, C E Dunn, R A Walford, E Heathcote, R Martin, A Pericleous, K Brzyska, K G Reid, M R Williams, N Wetherall, E McAleer, D Thomas, R Kiff, C Gouldthrope, S Milne, M J V Holmes, S Stokes, J Bartlett, J L ucas Carvalho, T Bloomfield, F Tongo, R H Bremner, N Yong, B A Atraszkiewicz, A Mehdi, M Tahir, G X J Sherliker, A K Tear, A Pandey, A Broyd, H M Omer, M Raphael, W W Chaudhry, S Shahidi, A S Jawad, C K Gill, I Hindle Fisher, I Adeleja, I J Clark, G E Aidoo-Micah, P W Stather, G J Salam, T E Glover, G Deas, N K Sim, R D Obute, W M Wynell-Mayow, M S Sait, N Mitha, G L Bernier, M Siddiqui, R Shaunak, A Wali, G Cuthbert, R Bhudia, E Webb, S Shah, N Ansari, M Perera, N Kelly, R McAllister, G H Stanley, C P Keane, V Shatkar, C Maxwell-Armstrong, L A Henderson, N Maple, R Manson, R D Adams, E Brown, E Semple, M Mills, A Daoub, A Marsh, A Ramnarine, J Hartley, M Malaj, P D Jewell, E A Whatling, N Hitchen, M Chen, B Goh, J Fern, S Rogers, L Derbyshire, D T Robertson, N Abuhussein, P Deekonda, A Abid, A Bakhsh, P L M Harrison, L Aildasani, H Turley, M A Sherif, G Pandey, J J Filby, A Johnston, E Burke, M Mohamud, K Gohil, A Y Tsui, R Singh, S J Lim, K O'Sullivan, L L McKelvey, S O'Neill, H F Roberts, F S Brown, Y Cao, R T Buckle, Y Liew, S Sii, C M Ventre, C J Graham, T Filipescu, A Yousif, R Dawar, A Wright, M Peters, R Varley, S Owczarek, S Hartley, M Khattak, A Iqbal, M Ali, B Durrani, Y Narang, G S Bethell, L Horne, R Pinto, K Nicholls, I Kisyov, H D Torrance, P Patel, M Patel, W English, S M Lakhani, S F Ashraf, M Venn, V Elangovan, Z Kazmi, J Brecher, S Sukumar, A Mastan, A Mortimer, J Parker, J Boyle, M Elkawafi, J Beckett, A Mohite, A Narain, E Mazumdar, A Sreh, A Hague, D Weinberg, L Fletcher, M Steel, H Shufflebotham, M Masood, Y Sinha, H Amin, C Jenvey, H Kitt, R Slade, A R Craig, C Deall, Y Sinha, J Gabriel, T Reakes, J Chervenkoff, E Strange, M O'Bryan, C Murkin, D Joshi, E Strange, T Bergara, S Naqib, D Wylam, E Strange, S E Scotcher, C M Hewitt, M T Stoddart, A Kerai, A J Trist, S J Cole, C L Knight, S Stevens, G E Cooper, R Ingham, J Dobson, J Wylie, A O'Kane, J Moradzadeh, A Duffy, C Henderson, S Ashraf, C McLaughin, T C Hoskins, R S Reehal, L R Bookless, R C McLean, E J Stone, E V Wright, H R Abdikadir, C Roberts, O Spence, M Srikantharajah, M Patel, E M Ruiz, J H Matthews, E Gardner, C Roberts, E Hester, P Naran, R Simpson, M Minhas, E Cornish, S A Semnani, D Rojoa, A Radotra, J Eraifej, K Eparh, D N E Smith, B D Mistry, S L Hickling, A Bhangu, W Din, C Liu, P Mithrakumar, V Mirdavoudi, M Rashid, C Mcgenity, O Hussain, M Kadicheeni, H Gardner, N Anim-Addo, J Pearce, A Aslanyan, C Ntala, T Sorah, J Parkin, M Alizadeh, A White, F Edozie, J Johnston, A Kahar, V Navayogaarajah, B Patel, D Carter, P Khonsari, A Burgess, B Patel, C Kong, A Ponweera, A Cody, Y Tan, A Y L Ng, A Croall, C Allan, S Ng, V Raghuvir, R Telfer, A D Greenhalgh, C N McKerr, M A Edison, B A Patel, K Dear, M R Hardy, P Williams, S Hassan, U Sajjad, E M O'Neill, S Lopes, L Healy, N Jamal, S Tan, D Lazenby, S B Husnoo, S Beecroft, T Sarvanandan, C Weston, N Bassam, S Rabinthiran, U Hayat, L Ng, D Varma, M Sukkari, A Mian, A Coates, A Omar, J W Kim, A Coates, J Sellathurai, J Mahmood, C O'Connell, R Bose, H Heneghan, P Lalor, J Matheson, C Doherty, C Cullen, D Cooper, S Angelov, C Drislane, A C D Smith, A Kreibich, E Palkhi, A Durr, A Lotfallah, D Gold, E Mckean, A Durr, A Dhanji, A Anilkumar, A Thacoor, A Durr, Z H Siddiqui, S Lim, A Piquet, S M Anderson, A Jindal, D R McCormack, J Gulati, A Ibrahim, A Jindal, S E Murray, S L Walsh, A McGrath, P Ziprin, E Y Chua, C N Lou, J Bloomer, H R Paine, D Osei-Kuffour, C J White, A Szczap, S Gokani, K Patel, M K Malys, A Reed, G E Torlot, E M Cumber, A Charania, S Ahmad, N Varma, H Cheema, L Austreng, H Petra, M Chaudhary, M I Zegeye, F Cheung, D Coffey, R S Heer, S Singh, E Seager, S Cumming, R S Suresh, S Verma, I B Ptacek, A M Gwozdz, T Yang, A A Khetarpal, S Shumon, T M P Fung, W Leung, P Kwang, L Chew, W Loke, A Curran, C Chan, C McGarrigle, K Mohan, S Cullen, E Wong, C Toale, D Collins, N Keane, B P Traynor, D Shanahan, A Yan, D J Jafree, C Topham, S Mitrasinovic, S Omara, B Gundogan, G Bingham, P M Lykoudis, B H Miranda, K Whitehurst, G Kumaran, Y Devabalan, H Aziz, M Shoa, S Dindyal, J A Yates, I Bernstein, G Rattan, J A Yates, R Coulson, S Stezaker, A Isaac, M Salem, A McBride, A Isaac, H McFarlane, L Yow, J MacDonald, R D Bartlett, S Turaga, U White, W Liew, N Yim, A Ang, A Simpson, D McAuley, E Craig, L Murphy, P Shepherd, J Y Kee, A Abdulmajid, A Chung, H L Warwick, A Livesey, P Holton, M D Theodoreson, S L Jenkin, J Turner, J H Entwisle, S T Marchal, S O'Connor, H K Blege, J M Aithie, L M Sabine, G E Stewart, S Jackson, A Kishore, C M Lankage, F Acquaah, H L Joyce, A Jindal, K L McKevitt, C J Coffey, A S Fawaz, K S Dolbec, D A O'Sullivan, J M Geraghty, E Lim, L Bolton, D FitzPatrick, C Robinson, T Ramtoola, S Collinson, L Grundy, P M McEnhill, G S Harbhajan Singh, D Loughran, D M Golding, R E Keeling, R P Williams, R D J Whitham, S Yoganathan, R Nachiappan, R J Egan, R Owasil, M L Kwan, A He, R W Goh, R Bhome, H Wilson, P J Teoh, K Raji, T Reeves, N Jayakody, J Matthams, J Chong, S Tan, C Y Luk, R J Greig, M Trail, G Charalambous, V Thirumal, A S Rocke, N Gardiner, F Bulley, N Warren, E Brennan, P Fergurson, R Wilson, H Whittingham, E J Brown, R Khanijau, K Gandhi, S Morris, A J Boulton, N Chandan, A E Barthorpe, R Maamari, S Sandhu, M McCann, L Higgs, V Balian, C Reeder, C Diaper, V Balian, T Sale, H Ali, V Balian, C H Archer, A K Clarke, J Heskin, P C Hurst, J D Farmer, L D O'Flynn, L Doan, B A Shuker, G D Stott, N A Vithanage, K A Hoban, P N Nesargikar, H R Kennedy, C M Grossart, E S M Tan, C S D Roy, P Sim, K E Leslie, D Sim, M H Abul, N Cody, A Y Tay, E Woon, S Sng, J Mah, J Robson, E Shakweh, V C Wing, H Mills, M M Li, T R Barrow, S Balaji, H E M Jordan, C Phillips, H Naveed, S Hirani, A Tai, R Ratnakumaran, A Sahathevan, A M A Shafi, M Seedat, R Weaver, A Batho, R Punj, H Selvachandran, N Bhatt, S Botchey, Z Khonat, K Brennan, K K Ong, C J Morrison, E Devlin, A Linton, E Galloway, S McGarvie, N Ramsay, H D McRobbie, H Whewell, W Dean, S Nelaj, M Eragat, A Mishra, T Kane, M Zuhair, M Wells, D Wilkinson, N Woodcock, E Sun, N Aziz, M K Abd Ghaffar

**Affiliations:** overall guarantor; University of Bristol, Bristol; Hull York Medical School, Hull; Leicester Medical School, Leicester; University of East Anglia, Norwich; University of Nottingham, Nottingham; University of Sheffield, Sheffield; Queen's University Belfast, Belfast; University of Limerick, Limerick; National University of Ireland, Galway; Royal College of Surgeons in Ireland, Dublin; Trinity College, Dublin; University College Cork, Cork; University College Dublin, Dublin; Imperial College, London; King's College, London; Queen Mary University, London; St George's University of London, London; University College London, London; University of Leeds, Leeds; University of Liverpool, Liverpool; University of Manchester, Manchester; Newcastle University Medical School, Newcastle upon Tyne; University of Aberdeen, Aberdeen; University of Dundee, Dundee; University of Edinburgh, Edinburgh; University of Glasgow, Glasgow; Brighton and Sussex Medical School, Brighton; Peninsula, Exeter and Plymouth; SouthamptonMedical School, Southampton; University of Cambridge, Cambridge; Cardiff University, Cardiff; University of Oxford, Oxford; Swansea University, Swansea; University of Birmingham, Birmingham; Keele University, Keele; University ofWarwick, Coventry; Aberdeen Royal Infirmary, Aberdeen; Addenbrooke's Hospital, Cambridge; Airedale General Hospital, Steeton; Alexandra Hospital, Redditch; Antrim Area Hospital, Antrim; Arrowe Park Hospital, Wirral; Barnet Hospital, Barnet; Basingstoke and North Hampshire Hospital, Basingstoke; Bedford Hospital, Bedford; Belfast City Hospital, Belfast; Blackpool Victoria Hospital, Blackpool; Bolton Royal Hospital, Bolton; Borders General Hospital, Melrose; Bradford Royal Infirmary, Bradford; Bristol Royal Infirmary, Bristol; Charing Cross Hospital, London; Chelsea and Westminster Hospital, London; Cheltenham General Hospital, Cheltenham; Chester Hospital, Chester; Colchester Hospital, Colchester; Conquest Hospital, Hastings; County Hospital, Stafford; Craigavon Area Hospital, Craigavon; Daisy Hill Hospital, Newry; Derriford Hospital, Plymouth; Dewsbury Hospital, Dewsbury; Diana Princess of Wales Hospital, Grimsby; Doncaster Royal Infirmary, Doncaster; Dumfries and Galloway Royal Infirmary, Dumfries; Ealing Hospital, Southall; Eastbourne District General Hospital, Eastbourne; East Surrey Hospital, Redhill; Epsom Hospital, Epsom; Forth Valley Royal Hospital, Larbert; Furness General Hospital, Barrow-in-Furness; Gartnavel General Hospital, Glasgow; George Eliot Hospital, Nuneaton; Glangwili General Hospital, Carmarthen; Glasgow Royal Infirmary, Glasgow; Gloucestershire Royal Hospital, Gloucester; Good Hope Hospital, Sutton Coldfield; Great WesternHospital, Swindon; Hammersmith Hospital, Hammersmith; Harrogate District Hospital, Harrogate; Heartlands Hospital, Birmingham; Hereford County Hospital, Hereford; Hillingdon Hospital, Uxbridge; Hinchingbrooke Hospital, Huntingdon; Homerton University Hospital, London; Huddersfield Royal Infirmary, Huddersfield; Hull Royal Infirmary, Hull, and Castle Hill Hospital, Cottingham; Inverclyde Royal Hospital, Greenock; Ipswich Hospital, Ipswich; James Connolly Hospital, Dublin; James Paget University Hospital, Great Yarmouth; Kent and Canterbury Hospital, Canterbury; King George Hospital, Ilford; King's College Hospital, London; King's Mill Hospital, Sutton-in-Ashfield; Kingston Hospital, Kingston upon Thames; Leicester Royal Infirmary, Leicester; Lincoln County Hospital, Lincoln; Lister Hospital, Stevenage; Manchester Royal Infirmary, Manchester; Mater Infirmorum Hospital, Belfast; Mayo General Hospital, Castlebar; Monklands Hospital, Airdrie; Morriston Hospital, Swansea; Musgrove Park Hospital, Taunton; New Cross Hospital, Wolverhampton; Newham University Hospital, London; Ninewells Hospital, Dundee; Nobles Hospital, Isle of Man; Norfolk and Norwich University Hospital, Norwich; Northern General Hospital, Sheffield; North Manchester General Hospital, Manchester; North Middlesex University Hospital NHS Trust, Edmonton; North Tyneside General Hospital, North Shields; Northwick Park Hospital, Harrow; Nottingham City Hospital, Nottingham; John Radcliffe and Churchill Hospitals, Oxford; Perth Royal Infirmary, Perth; Peterborough City Hospital, Peterborough; Portsmouth Hospitals NHS Trust Queen Alexandra Hospital, Portsmouth; Prince Charles Hospital, Merthyr Tydfil; Princess of Wales Hospital, Bridgend; Princess Royal Hospital, Haywards Heath; Princess Royal University Hospital, Bromley; Queen Elizabeth Hospital, Birmingham; Queen Elizabeth Hospital, King's Lynn; Queen Elizabeth Hospital, Woolwich; Queen's Hospital, Romford; Queen's Medical Centre, Nottingham; Raigmore Hospital, Inverness; Rotherham General Hospital, Rotherham; Royal Berkshire Hospital, Reading; Royal Blackburn Hospital, Blackburn; Royal Devon and Exeter Hospital, Exeter; Royal Glamorgan Hospital, Ynysmaerdy; Royal Gwent Hospital, Newport; Royal Infirmary of Edinburgh, Edinburgh; Royal Lancaster Infirmary, Lancaster; Royal Liverpool Hospital, Liverpool; Royal London Hospital, London; Royal Preston Hospital, Preston; Royal Shrewsbury Hospital, Shrewsbury; Royal Stoke University Hospital, Stoke-on-Trent; Royal Sussex County Hospital, Brighton; Royal United Hospital, Bath; Royal Victoria Hospital, Belfast; Royal Victoria Infirmary, Newcastle upon Tyne; Russell's Hall Hospital, Dudley; Salford Royal Hospital, Salford; Sandwell General Hospital, West Bromwich; Scarborough Hospital, Scarborough; Scunthorpe Hospital, Scunthorpe; Southend Hospital, Westcliff-on-Sea; Southern General Hospital, Glasgow; Southmead Hospital, Bristol; Southport Hospital, Southport; South Tipperary Hospital, Tipperary; Stepping Hill Hospital, Stockport; St George's Hospital, London; St Helier Hospital, Carshalton; St James’ Hospital, Dublin; St James's University Hospital, Leeds; St Luke's Hospital, Limerick; St Mary's Hospital, London; Stoke Mandeville Hospital, Aylesbury, and High Wycombe Hospital, High Wycombe; St Peter's Hospital, Chertsey; St Thomas’ Hospital, London; Sunderland Royal Hospital, Sunderland; Tameside General Hospital, Ashton-under-Lyne; The Adelaide and Meath Hospital, Dublin; The Royal Free Hospital, London; The Whittington Hospital, London; Tullamore Hospital, Tullamore; Ulster Hospital, Belfast; University College London Hospital, London; University Hospital Ayr, Ayr; University Hospital Coventry and Warwickshire, Coventry; University Hospital Crosshouse, Crosshouse; University Hospital Lewisham, Lewisham; University Hospital Limerick, Limerick; University Hospital of South Manchester, Manchester; University Hospital of Wales, Cardiff; University Hospital Southampton NHS Foundation Trust, Southampton; Victoria Hospital, Kirkcaldy; Victoria Infirmary, Glasgow; Walsall Manor Hospital, Walsall; Warrington Hospital, Warrington; Warwick Hospital, Warwick; Western General Hospital, Edinburgh; Western Infirmary, Glasgow; West Middlesex Hospital, Isleworth; Whipps Cross Hospital, London; Whiston Hospital, Prescot; Wishaw General Hospital, Wishaw; Wrexham Maelor Hospital, Wrexham; York Hospital, York; Ysbyty Gwynedd, Bangor

## Abstract

**Background:**

There is currently conflicting evidence surrounding the effects of obesity on postoperative outcomes. Previous studies have found obesity to be associated with adverse events, but others have found no association. The aim of this study was to determine whether increasing body mass index (BMI) is an independent risk factor for development of major postoperative complications.

**Methods:**

This was a multicentre prospective cohort study across the UK and Republic of Ireland. Consecutive patients undergoing elective or emergency gastrointestinal surgery over a 4-month interval (October–December 2014) were eligible for inclusion. The primary outcome was the 30-day major complication rate (Clavien–Dindo grade III–V). BMI was grouped according to the World Health Organization classification. Multilevel logistic regression models were used to adjust for patient, operative and hospital-level effects, creating odds ratios (ORs) and 95 per cent confidence intervals (c.i.).

**Results:**

Of 7965 patients, 2545 (32·0 per cent) were of normal weight, 2673 (33·6 per cent) were overweight and 2747 (34·5 per cent) were obese. Overall, 4925 (61·8 per cent) underwent elective and 3038 (38·1 per cent) emergency operations. The 30-day major complication rate was 11·4 per cent (908 of 7965). In adjusted models, a significant interaction was found between BMI and diagnosis, with an association seen between BMI and major complications for patients with malignancy (overweight: OR 1·59, 95 per cent c.i. 1·12 to 2·29, *P* = 0·008; obese: OR 1·91, 1·31 to 2·83, *P* = 0·002; compared with normal weight) but not benign disease (overweight: OR 0·89, 0·71 to 1·12, *P* = 0·329; obese: OR 0·84, 0·66 to 1·06, *P* = 0·147).

**Conclusion:**

Overweight and obese patients undergoing surgery for gastrointestinal malignancy are at increased risk of major postoperative complications compared with those of normal weight.

## Teaser

High BMI increases risk of complications

## Introduction

The incidence of obesity is rising rapidly across high-income countries, with the current prevalence in the USA (36 per cent) and UK (26 per cent) expected to double by 2050^1^. It is estimated that up to 66 per cent of patients undergoing surgery in the UK are overweight^2^. Current evidence is conflicting regarding the impact of obesity on postoperative complications after major surgery. Contemporary multicentre studies^3–7^ in specific patient groups from Japan, Denmark, Switzerland and the USA have associated obesity with worse or neutral short-term postoperative outcomes. A recent risk-adjusted analysis from the US National Surgical Quality Improvement Program (NSQIP) identified an ‘obesity paradox’ in non-bariatric surgery, whereby overweight and obese patients had a lower adjusted risk of postoperative mortality^8^.

With at least 600 000 major gastrointestinal operations being carried out each year in the UK, knowing whether obesity increases postoperative complication rates is important for patients, doctors and commissioners^9^. If increasing body mass is associated with worse outcomes, patients may benefit from perioperative optimization. Depending on the type and timing of surgery, this may include nutritional optimization before surgery, the application of beneficial technology (such as minimally invasive surgery) and access to high-dependency postoperative care. Firm evidence would provide justification for research to assess these programmes, as there may be unintended consequences, including malnutrition, associated with weight loss.

As the variable of interest is body mass index (BMI), randomized trials assigning patients to subgroups of interest (normal weight, overweight and obese) are not possible. Current evidence is thus based on analysis of observational data. A dedicated, prospective analysis in a broad group of patients undergoing elective and emergency surgery with a preplanned, detailed risk adjustment strategy is lacking. This prospective study aimed to determine associations between BMI and postoperative morbidity following elective and emergency major gastrointestinal surgery in the UK and Ireland.

## Methods

The protocol for this multicentre prospective cohort study was disseminated through a multinational medical student and trainee surgical collaborative network (with coverage in the UK and Republic of Ireland)^10^. This network has been described in detail elsewhere^11–13^. Briefly, teams of medical students with senior registrar and consultant oversight collected data on consecutive patients across 2-week periods. Results are reported according to Strengthening the Reporting of Observational Studies in Epidemiology (STROBE) and Statistical Analyses and Methods in the Published Literature (SAMPL) guidelines^14,15^. National Research Ethics Service review of the protocol deemed that full ethical review was not required owing to the observational and anonymous nature of this study. Each participating centre was responsible for local registration as service evaluation or clinical audit. In the Republic of Ireland, each participating centre was responsible for completing the research ethics process at their centre, as required by local guidelines.

### Eligibility criteria

Consecutive adult patients (aged at least 18 years) undergoing gastrointestinal or hepatobiliary surgery were included in the study. At each centre a minimum 2-week interval was selected for patient inclusion between 1 October and 12 November 2014; multiple non-overlapping 2-week periods per hospital were allowed. Eligible procedures were those involving surgery on any part of the gastrointestinal tract or biliary tree, including a hospital admission with an overnight stay. Both elective and emergency procedures performed using open, laparoscopic, laparoscopically assisted or robotic approaches were included. Patients undergoing day-case, urological, gynaecological, vascular or transplant procedures were excluded.

### Outcome measures

The Clavien–Dindo system was used to define postoperative complications, whereby complication severity is defined by the subsequent treatment required^16^. This classification system is a validated means to determine the severity of postoperative complications based on the treatment received. All postoperative events were included, even when there was no direct relationship with the surgery. Each patient's highest Clavien–Dindo grade complication was recorded. The primary outcome measure was the 30-day major complication rate (Clavien–Dindo grade III–V), which included endoscopic, radiological or surgical reintervention (Clavien-Dindo III), unexpected critical care admission (Clavien–Dindo IV) and death (Clavien–Dindo V). The secondary outcome was the surgical-site infection (SSI) rate, chosen as it has been associated with obesity previously^6^, defined by the Centers for Disease Control definition^17^. Additional secondary outcome measures listed in the protocol included: outcomes for underweight patients, system-specific complications, unplanned admission to the critical care unit, reoperation and readmission. For the purposes of clarity, these will be described in detail in further reports.

### Main explanatory variable

The main explanatory variable was preoperative BMI, assessed either in the preoperative assessment clinic or on admission. This was calculated as weight (in kilograms) divided by height (in metres) squared. As the primary aim of this study was to assess the effect of being overweight or obese, patients were stratified by BMI according to groups defined by the World Health Organization (WHO): normal weight (BMI 18·5–24·9 kg/m^2^), overweight (BMI 25·0–29·9 kg/m^2^) and obese (BMI at least 30·0 kg/m^2^)^18^.

### Explanatory variables

Explanatory variables were collected in order to provide a risk-adjusted estimate. Variables were predefined and selected on the basis of clinical plausibility. The following patient- and operation-level factors were selected.

#### Patient

To account for co-morbidities, the American Society of Anesthesiologists (ASA) fitness grade was recorded and the Revised Cardiac Risk Index (RCRI) calculated for each patient. The ASA grade takes into account disease severity and is a reliable metric for the measurement of postoperative mortality and complications^19^. The RCRI is used to estimate a patient's risk of perioperative cardiac complications, including cardiac death, non-fatal myocardial infarction and non-fatal cardiac arrest^20^. Age, sex and smoking status were also collected.

#### Operation

A novel Hospital Episode Statistics (HES)-based operative risk score was devised in order to determine the mortality risk associated with each specific operation. HES are summary, population-level data available from National Health Service administrative records. From this aggregate data, a summary of mortality rates by procedure during 2009 and 2010 was obtained as the most recent available, procedure-level data. Procedures included in the study were identified and classified according to their 30-day mortality rate into predefined strata of low-risk (less than 1 per cent), moderate-risk (1– 9·9 per cent) and high-risk (10 per cent or more) groups (*Table S1*, supporting information). Additionally, diagnosis (benign *versus* malignant), urgency of surgery (elective *versus* emergency) and operative approach (open *versus* laparoscopic) were included.

### Data accuracy

Before data collection, all collaborators were invited to attend investigator meetings and complete a mandatory online training module. To ensure high data quality, only submitted data sets with over 95 per cent completeness for both case ascertainment and the study data fields were eligible for inclusion. If a large proportion of data was missing, data would be imputed using Markov chain Monte Carlo equations. In addition, a process of data validation was performed by independent collaborators. Ten per cent of all included patients were validated independently for accuracy. Twelve key predefined data points were validated for each patient (age, sex, height, weight, Index of Multiple Deprivation decile, Malnutrition Universal Screening Tool score, timing of BMI measurement, urgency of operation, postoperative critical care admission, complications, return to theatre, readmission). A data point was defined as a single value in each of the data fields for an individual patient. The overall accuracy was calculated according to number of correct validated data points divided by the total number of validated data points. The case ascertainment rate was determined by independent review of theatre logbooks for eligible cases, cross-referenced against the number of actual cases submitted.

### Data handling

Data collection was performed using the secure research electronic capture database (REDCap) system^21^. The submitted data were then checked centrally and, where missing data were identified, the local investigator was contacted and asked to complete the record. Once vetted, the record was accepted into the data set for analysis.

### Sample size

This study was powered to detect a minimum significant difference between obese and normal-weight patients, although no upper limit on patient numbers was set owing to the nature of the study. A minimum of 3550 normal-weight and obese patients would provide 80 per cent power to detect a 2·8 per cent increase in major postoperative complication rate from 8 to 10·8 per cent (α = 0·05). This was based on previous estimates and a complication rate increase that would be deemed clinically significant^11^.

### Statistical analysis

There was likely to be considerable selection bias in routine practice that influenced the crude outcome of this study. This is related to individual patient risk factors and the risks of the operation they are undergoing (for example obese patients may have higher risk owing to co-morbidity; equally they may have better outcome after lower-risk bariatric surgery). Additionally, there may be bias in how patients are treated across hospitals. Multilevel models were used to determine unbiased distributions of fixed-effect regression coefficients for the outcomes of major complication rate (primary outcome measure) and SSI (secondary outcome measure). The hospital was considered as a single, level 1 random effect, with individual patient and operative risks entered as level 2 fixed effects. Age was expressed as a continuous variable with corresponding odds ratios (ORs) relating to a per-year increase. Explanatory variables contained within statistical models were selected based on clinical plausibility and independence, and model selection was informed using the Akaike information criterion. Effect estimates are presented as ORs and bootstrapped 95 per cent confidence intervals, and statistical significance expressed as *P* values. ORs were generated to describe the relationships between BMI groups and the outcome of interest. An OR greater than 1 represented increased likelihood in the experimental group (obese) *versus* the control group (normal BMI). The OR describes the relationship between an explanatory variable and an outcome in terms of odds of suffering a complication, rather than the risk. The OR was considered to be statistically significant at the *P* < 0·050 level. All relevant second-order interactions were examined. Differences between categorical demographic groups were tested using the Kruskal–Wallis test or Welch's *t* test for continuous data or χ^2^ test for proportions. Two-sided statistical significance was defined at the level of *P* < 0·050. Data analysis was undertaken using R Foundation Statistical software (R 3.2.1) with the Hmisc, ggplot2, plyr, lme4, reshape2, RCurl, splines and stringr packages (R Foundation for Statistical Computing, Vienna, Austria).

## Results

**Fig. 1:**
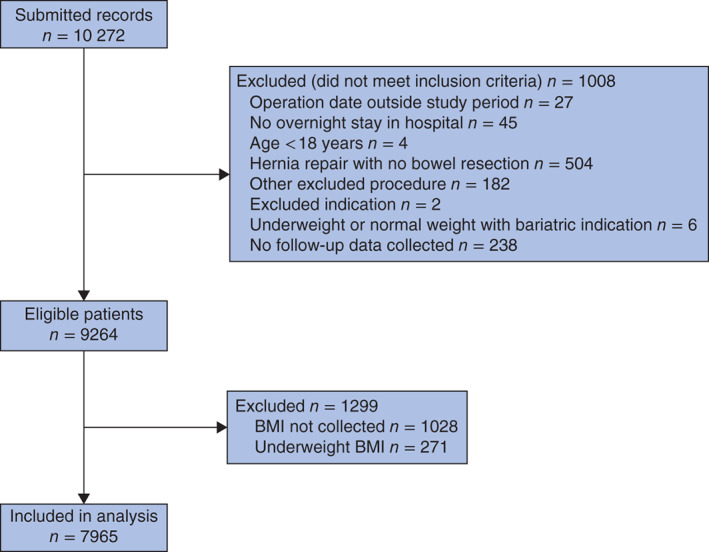
Flow chart of patient inclusion

Of 10 272 records submitted from 163 centres, 9264 eligible records were submitted to the final analysis (*Fig. 1*). Of these, 7965 (86·0 per cent) had a preoperative BMI measurement; 2545 patients (32·0 per cent) were of normal weight, 2673 (33·6 per cent) were overweight and 2747 (34·5 per cent) were obese. Independent validation of 1008 patients with 12 096 data points showed that the accuracy was 98·0 per cent and the case ascertainment rate 92·2 per cent.

### Demographics

There were significant differences in patient characteristics across BMI groups (*Table 1*). Patients in the overweight group were older and patients in obese group were younger than those of normal weight; both groups were more likely to undergo lower-risk surgery and more likely to be operated on using a laparoscopic approach. Obese and overweight patients had higher ASA and RCRI scores, reflecting greater co-morbidity, but normal-weight patients underwent proportionally more emergency and higher-risk procedures. The missing data were minimal and therefore imputation was not required.

**Table 1:** Baseline demographics and operative details by body mass index group

	Normal (*n* = 2545)	Overweight (*n* = 2673)	Obese (*n* = 2747)	*P* ^†^
Age (years)*	55·3(28·2)	57·8(17·7)	53·3(16·2)	< 0·001^‡^
Sex				< 0·001
M	1164 (45·7)	1451 (54·3)	1035 (37·7)	
F	1381 (54·3)	1222 (45·7)	1712 (62·3)	
ASA fitness grade				< 0·001
I	793 (31·2)	780 (29·2)	549 (20·0)	
II	1106 (43·5)	1282 (48·0)	1441 (52·5)	
III	522 (20·5)	514 (19·2)	681 (24·8)	
IV	91 (3·6)	74 (2·8)	57 (2·1)	
V	17 (0·7)	5 (0·2)	5 (0·2)	
Missing	16 (0·6)	18 (0·7)	14 (0·5)	
Smoking status				< 0·001
Non-smoker	1998 (78·5)	2245 (84·0)	2315 (84·3)	
Current smoker	547 (21·5)	428 (16·0)	432 (15·7)	
Revised Cardiac Risk Index				< 0·001
0	2059 (80·9)	2006 (75·0)	1942 (70·7)	
I	366 (14·4)	488 (18·3)	607 (22·1)	
≥ II	120 (4·7)	177 (6·6)	196 (7·1)	
Missing	0 (0)	2 (0·1)	2 (0·1)	
Operative risk class				< 0·001
Low	966 (38·0)	1100 (41·2)	1490 (54·2)	
Moderate	848 (33·3)	883 (33·0)	675 (24·6)	
High	731 (28·7)	690 (25·8)	582 (21·2)	
Diagnosis				< 0·001
Benign	1803 (70·8)	1843 (68·9)	1796 (65·4)	
Malignant	742 (29·2)	830 (31·1)	557 (20·3)	
Bariatric	0 (0)	0 (0)	394 (14·3)	
Urgency of surgery				< 0·001
Elective	1364 (53·6)	1660 (62·1)	1901 (69·2)	
Emergency	1180 (46·4)	1012 (37·9)	846 (30·8)	
Missing	1 (0·0)	1 (0·0)	0 (0)	
Operative approach				< 0·001
Open	1163 (45·7)	1094 (40·9)	834 (30·4)	
Laparoscopic	1379 (54·2)	1578 (59·0)	1911 (69·6)	
Missing	3 (0·1)	1 (0·0)	2 (0·1)	

Values in parentheses are percentages unless indicated otherwise;

* values are mean(s.d.). ASA, American Society of Anesthesiologists.

† χ^2^ test, except

‡ Kruskal–Wallis test.

### Case mix

Overall, malignancy was the most common diagnosis, followed by appendicitis and gallstone disease (*Table 2*). In the overweight and obese groups, more procedures were undertaken for gallstone disease than in the normal-weight group. Of the obese patients, 14·3 per cent underwent bariatric surgery. Obese patients were more likely to have laparoscopic surgery (69·6 *versus* 54·2 per cent of normal-weight patients), which corresponded with overweight and obese patients undergoing less risky procedures than normal-weight patients. However, obese patients were still subjected to high-risk surgery, with 30·8 per cent undergoing emergency surgery and 45·8 per cent a moderate- or high- risk operative procedure (*Table 1*).

**Table 2:** Summary of diagnoses by body mass index group

	Normal (*n* = 2545)	Overweight (*n* = 2673)	Obese (*n* = 2747)
Malignancy	742 (29·2)	830 (31·1)	557 (20·3)
Appendicitis	623 (24·5)	508 (19·0)	385 (14·0)
All other indications	406 (16·0)	332 (12·4)	245 (8·9)
Cholecystitis	299 (11·7)	558 (20·9)	791 (28·8)
Inflammatory bowel disease	207 (8·1)	123 (4·6)	77 (2·8)
Diverticulitis	47 (1·8)	75 (2·8)	65 (2·4)
Peptic ulcer disease	46 (1·8)	30 (1·1)	17 (0·6)
Ischaemic bowel	39 (1·5)	29 (1·1)	18 (0·7)
Other liver or pancreatic disease	29 (1·1)	47 (1·8)	56 (2·0)
Hernia	25 (1·0)	31 (1·2)	38 (1·4)
Pancreatitis	24 (0·9)	27 (1·0)	34 (1·2)
Faecal perforation	21 (0·8)	18 (0·7)	14 (0·5)
Gastro-oesophageal reflux	20 (0·8)	48 (1·8)	39 (1·4)
Fistula	17 (0·7)	17 (0·6)	17 (0·6)
Bariatric indication	0 (0)	0 (0)	394 (14·3)

Values in parentheses are percentages.

### Outcomes

For all patients, the overall 30-day major (Clavien–Dindo III–V) complication rate was 11·4 per cent (908 of 7965 patients). The overall SSI rate was 5·9 per cent (472 of 7965), increasing to 11·1 per cent (90 of 808) in those undergoing emergency colorectal resection. An unadjusted breakdown of these outcomes by BMI group is shown in *Table 3*.

**Table 3:** Unadjusted 30-day complication rates by body mass index group

	Normal (*n* = 2545)	Overweight (*n* = 2673)	Obese (*n* = 2747)	*P* *
Major complications (Clavien–Dindo III–V)				0·040
No	2238 (87·9)	2351 (88·0)	2468 (89·8)	
Yes	307 (12·1)	322 (12·0)	279 (10·2)	
Surgical-site infection				0·274
No	2410 (94·7)	2507 (93·8)	2576 (93·8)	
Yes	135 (5·3)	166 (6·2)	171 (6·2)	

Values in parentheses are percentages.

* χ^2^ test.

**Fig. 2:**
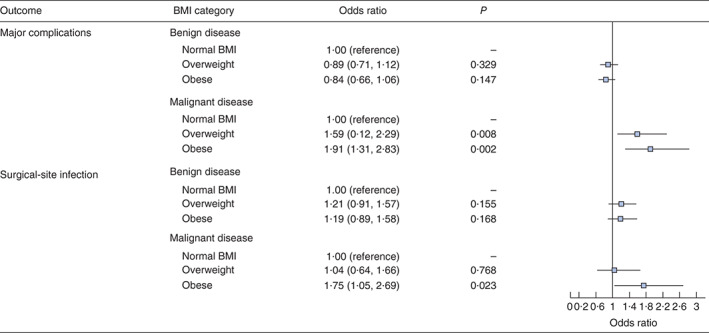
Analysis of the interaction between body mass index (BMI) and diagnosis using recoded variables. Odd ratios are shown with 95 per cent confidence intervals

A significant interaction was found between BMI and malignant/benign diagnosis for major complications (*Table 4*) and for SSI (*Table 5*). Taking these interactions into account produced adjusted ORs for each group (*Fig. 2*). Overweight and obese patients with malignancy were at greater risk of major complications. Obese patients with malignancy were at greater risk of SSI. There were no associations between any BMI groups with a benign diagnosis and either outcome.

**Table 4:** Univariable and multilevel logistic regression analyses to determine association of 30-day major postoperative complications (Clavien–Dindo III–V) with patient and operative factors

	Univariable analysis		Multilevel analysis	
	Odds ratio	*P*	Odds ratio	*P*
BMI				
Normal	1·00 (reference)	–	1·00 (reference)	–
Overweight	1·00 (0·85, 1·18)	0·985	0·89 (0·71, 1·12)*	0·329
Obese	0·82 (0·69, 0·98)	0·027	0·84 (0·66, 1·06)*	0·147
Age (per year)	1·02 (1·02, 1·03)	< 0·001	1·01 (1·00, 1·01)	< 0·001
ASA fitness grade				
I–II	1·00 (reference)	–	1·00 (reference)	–
III–V	2·82 (2·44, 3·25)	< 0·001	1·97 (1·66, 2·33)	< 0·001
Diagnosis				
Benign	1·00 (reference)	–	1·00 (reference)	–
Malignant	1·89 (1·64, 2·18)	< 0·001	0·77 (0·58, 1·02)	0·064
Revised Cardiac Risk Index				
0	1·00 (reference)	–	1·00 (reference)	–
I	1·68 (1·42, 1·98)	< 0·001	1·08 (0·90, 1·31)	0·421
≥ II	2·21 (1·73, 2·79)	< 0·001	1·14 (0·85, 1·46)	0·346
Operative risk class				
Low	1·00 (reference)	–	1·00 (reference)	–
Moderate	3·67 (3·06, 4·41)	< 0·001	3·05 (2·43, 3·79)	< 0·001
High	3·52 (2·92, 4·27)	< 0·001	2·74 (2·24, 3·40)	< 0·001
Sex				
M	1·00 (reference)	–	1·00 (reference)	–
F	0·66 (0·57, 0·76)	< 0·001	0·77 (0·67, 0·89)	< 0·001
Smoking status				
Non-smoker	1·00 (reference)	–	1·00 (reference)	–
Current smoker	1·00 (0·83, 1·19)	0·971	1·06 (0·86, 1·30)	0·548
Timing of surgery				
Elective	1·00 (reference)	–	1·00 (reference)	–
Emergency	1·13 (0·98, 1·30)	0·087	1·64 (1·39, 1·94)	< 0·001
Interaction variables BMI group by diagnosis				
Overweight by malignancy	–	–	1·59 (1·12, 2·29)^†^	0·008
Obese by malignancy	–	–	1·91 (1·31, 2·83)^†^	0·002

Values in parentheses are 95 per cent confidence intervals. An unexpected interaction was identified between body mass index (BMI) and diagnosis. This is included in the multilevel logistic regression model. The interaction can be interpreted as follows:

* the exponentiated coefficients for BMI categories overweight and obese given in the model including the interaction term represent the odds ratios where the diagnosis is benign.

† The exponentiated coefficients for the interaction terms can be thought of as a ratio of odds ratios. Akaike information criterion 5164·07. ASA, American Society of Anesthesiologists.

**Table 5:** Univariable and multilevel logistic regression analyses to determine association of surgical-site infection with patient and operative factors

	Univariable analysis		Multilevel analysis	
	Odds ratio	*P*	Odds ratio	*P*
BMI				
Normal	1·00 (reference)	–	1·00 (reference)	–
Overweight	1·18 (0·94, 1·50)	0·161	1·21 (0·91, 1·57)*	0·155
Obese	1·19 (0·94, 1·50)	0·152	1·19 (0·89, 1·58)*	0·168
Age (per year)	1·01 (1·00, 1·01)	0·007	1·00 (1·00, 1·01)	0·496
ASA fitness grade				
I–II	1·00 (reference)	–	1·00 (reference)	–
III–V	1·36 (1·10, 1·66)	0·003	0·98 (0·79, 1·22)	0·903
Diagnosis				
Benign	1·00 (reference)	–	1·00 (reference)	–
Malignant	1·51 (1·24, 1·84)	< 0·001	0·88 (0·60, 1·30)	0·430
Revised Cardiac Risk Index				
0	1·00 (reference)	–	1·00 (reference)	–
I	1·42 (1·13, 1·78)	0·002	1·24 (0·97, 1·57)	0·083
≥ II	1·75 (1·24, 2·40)	0·001	1·42 (0·96, 1·96)	0·081
Operative risk class				
Low	1·00 (reference)	–	1·00 (reference)	–
Moderate	2·23 (1·77, 2·83)	< 0·001	2·42 (1·82, 3·21)	< 0·001
High	2·48 (1·96, 3·15)	< 0·001	2·56 (1·98, 3·31)	< 0·001
Sex				
M	1·00 (reference)	–	1·00 (reference)	–
F	0·76 (0·63, 0·92)	0·005	0·86 (0·72, 1·04)	0·132
Smoking status				
Non-smoker	1·00 (reference)	–	1·00 (reference)	–
Current smoker	1·22 (0·96, 1·53)	0·091	1·29 (1·00, 1·63)	0·047
Timing of surgery				
Elective	1·00 (reference)	–	1·00 (reference)	–
Emergency	1·09 (0·90, 1·31)	0·383	1·47 (1·20, 1·80)	< 0·001
Interaction variables BMI group by diagnosis				
Overweight by malignancy	–	–	1·04 (0·64, 1·66)^†^	0·768
Obese by malignancy	–	–	1·75 (1·05, 2·69)^†^	0·023

Values in parentheses are 95 per cent confidence intervals. An unexpected interaction was identified between body mass index (BMI) and diagnosis. This is included in the multilevel logistic regression model. The interaction can be interpreted as follows:

* the exponentiated coefficients for BMI categories overweight and obese given in the model including the interaction term represent the odds ratios where the diagnosis is benign.

† The exponentiated coefficients for the interaction terms can be thought of as a ratio of odds ratios. Akaike information criterion 3454·58. ASA, American Society of Anesthesiologists.

To explore the interaction between diagnosis and obesity, a further analysis of obese patients alone was performed (*Table 6*). This showed that obese patients undergoing surgery for malignancy were subject to higher patient- and operation-level risks than obese patients undergoing surgery for benign conditions. This included older age, higher ASA grades, higher RCRI scores, more open surgery, and a greater proportion of patients undergoing high-risk surgical procedures (18·3 per cent benign *versus* 32·7 per cent malignant). A comparison of patients undergoing surgery for malignancy only revealed that those who were obese had greater risk in terms of ASA grade and RCRI score than normal-weight or overweight patients (*Table S2*, supporting information). Obese patients undergoing laparoscopic surgery for malignancy were almost twice as likely to require conversion to an open operation than normal-weight and overweight patients, and obese patients having surgery for a benign condition. Patient characteristics and outcomes within the obese group according to WHO subgroups are described in *Table S3* (supporting information). The operative risk class stratification predicted increasing mortality across all three BMI categories (*Table S4*, supporting information).

**Table 6:** Characteristics and outcomes of obese patients by benign or malignant diagnosis

	Benign (*n* = 2190)	Malignant (*n* = 557)	*P* ^†^
Age (years)*	50·2(15·8)	65·4(11·5)	< 0·001^‡^
Sex			< 0·001
M	701 (32·0)	334 (60·0)	
F	1489 (68·0)	223 (40·0)	
ASA fitness grade			< 0·001
I	505 (23·1)	44 (7·9)	
II	1143 (52·2)	298 (53·5)	
III	485 (22·1)	196 (35·2)	
IV	40 (1·8)	17 (3·1)	
V	5 (0·2)	0 (0·0)	
Missing	12 (0·5)	2 (0·4)	
Smoking status			0·007
Non-smoker	1825 (83·3)	490 (88·0)	
Current smoker	365 (16·7)	67 (12·0)	
Revised Cardiac Risk Index			< 0·001
0	1624 (74·2)	318 (57·1)	
I	429 (19·6)	178 (32·0)	
≥ II	135 (6·2)	61 (11·0)	
Missing	2 (0·1)	0 (0)	
Operative risk class			< 0·001
Low	1464 (66·8)	26 (4·7)	
Moderate	326 (14·9)	349 (62·7)	
High	400 (18·3)	182 (32·7)	
Urgency of surgery			< 0·001
Elective	1385 (63·2)	516 (92·6)	
Emergency	805 (36·8)	41 (7·4)	
Operative approach			< 0·001
Open	365 (16·7)	287 (51·5)	
Open, laparoscopy-assisted	33 (1·5)	34 (6·1)	
Laparoscopy	1665 (76·0)	179 (32·1)	
Laparoscopy converted to open	126 (5·8)	56 (10·1)	
Missing	1 (0·0)	1 (0·2)	
Outcomes			
Major complications (Clavien–Dindo III–V)			< 0·001
No	2019 (92·2)	449 (80·6)	
Yes	171 (7·8)	108 (19·4)	
Surgical-site infection			< 0·001
No	2081 (95·0)	495 (88·9)	
Yes	109 (5·0)	62 (11·1)	

Values in parentheses are percentages unless indicated otherwise;

* values are mean(s.d.). ASA, American Society of Anesthesiologists.

† χ^2^ test, except

‡ Welch's *t* test.

## Discussion

This large, prospective study found that overweight and obese patients undergoing surgery for gastrointestinal malignancy were at increased risk of major postoperative complications compared with normal-weight patients. Overweight and obese patients undergoing surgery for benign conditions did not have higher risks of complications, nor was body mass associated with adverse outcomes in this group. Obese patients were at higher risk of SSI overall, with a weak association for overweight patients.

These findings may be explained by differences in the characteristics of patients undergoing surgery for either a benign or malignant diagnosis. Because of the urgency of their treatment, patients having surgery for malignancy are not readily selected or operations delayed based on fitness. However, those undergoing surgery for benign conditions are likely to be subject to a selection bias of fitter patients for generally lower-risk procedures. Obese patients underwent far more bariatric and gallbladder operations, leading to a higher number of laparoscopic and elective procedures. In malignant disease, overweight and obese patients had more co-morbidity than normal-weight patients, which may have contributed to their increased risk. Less use of laparoscopy and higher rates of conversion in obese patients with malignancy also suggests technical difficulties. Although these risks were adjusted for in the present models, it is possible that the variables included did not fully adjust for the risk to which these patients were subjected.

The main strength of this study is the accurate and comprehensive risk adjustment from a validated prospective data set, which did not rely on administrative or retrospective data. One of the most important clinical factors affecting mortality risk is the magnitude of the operation performed and its timing. To account for this, a novel HES-based operative risk score was developed, which can be used by future investigators to stratify their own patient groups.

The main limitation of this study is the presence of selection bias between indication for surgery and obesity. Here, morbidly obese patients with benign conditions and co-morbidities might not have been treated with surgery, as clinicians may have opted for an alternative management plan or weight loss owing to the perceived risk of higher complications. Thus surgery for benign conditions still needs to be balanced against the risks when co-morbidities are present. Furthermore, patients who received a benign diagnosis may in fact have had malignant disease. In the elective setting, this is highly unlikely, as malignant disease is staged before operation and confirmed histologically before surgery. The group of patients undergoing emergency surgery may have been at higher risk of a misclassified diagnosis as they may not have received preoperative histopathology, but were likely to have had cross-sectional imaging. Nevertheless, as histopathology reports are usually available within 30 days of operation and therefore the follow-up period, the risk of this influencing the results was low. Complications occurring after discharge that did not re-present to the same hospital may not have been detected, but are of uncertain consequence. The 30-day follow-up period was selected to ensure that the study was logistically feasible compared with a longer follow-up period which may have captured additional complications. Despite this, 30-day follow-up remains a sensitive measure and is known to correlate well with 90-day outcomes^22^. Finally, an independent validation process proved the data to be both highly accurate and complete.

Previous population-level studies have suggested an ‘obesity paradox’, whereby overweight and obese patients have lower postoperative complication rates than those of normal weight^8^. The present findings refute this, as obese patients with malignant disease were at significantly higher risk of major complications. There are four possible explanations for this difference. It may reflect different statistical risk adjustment strategies. Here, the risk of type I errors was limited by fitting models according to prespecified clinically relevant factors. The novel HES-based risk score provides a high level of quality control for impact of operation, which was lacking in other studies. Other factors were adjusted for including: age, disease severity (ASA grade), urgency of operation and smoking status. A further reason why the present finding has not been observed in previous studies is that there may have been selection bias in other populations, with obese patients being denied surgery or having surgery delayed. It is still reasonable to suggest that there may be other physiological or lifestyle differences between this UK and Irish population compared with previously described North American populations, which remain unaccounted for. Different preoperative optimization practices may also exist, especially with the depth of community care from general practitioners in the UK. Finally, treatment differences in UK and Irish practice that were not captured in this study may be associated with different complications, such as the use of neoadjuvant therapies for cancer.

This large study from the UK and Ireland is globally relevant, with its findings potentially affecting hundreds of thousands of patients undergoing major gastrointestinal surgery every year. Within this context, increased use of laparoscopy may reduce complications and SSI. Therefore, a laparoscopic-first approach should be adopted routinely for all overweight and obese patients where possible^23^.

Further research is required to determine cost-effective methods to reduce perioperative complications in overweight and obese patients with malignancy. In the present study, patients undergoing surgery for malignancy were at an increased risk of complications; therefore this is a specifically high-risk group. New ways of preoperative optimization of patients to reduce complications, such as preoperative cardiopulmonary exercise, have shown promise in pilot studies. Within the obese and overweight patient population, improving fitness for surgery may prove a useful way of reducing major complications^24,25^. In light of the present findings, obese and overweight patients should be a key stakeholder group for the development of these studies. Preoperative weight-loss programmes may not be an ideal solution, as a poor catabolic nutritional state is associated with worse postoperative outcomes and heightened mortality^26^. During the operation, the physiological differences found between normal and obese patients should be considered carefully, particularly with regard to fluid management and drug doses tailored toward achieving sufficient tissue concentrations. Intraoperative hypothermia has been shown to increase the risk of SSI^27^; obese or overweight patients should therefore receive perioperative warming because of their greater surface area. Finally, adequate fluid management in obese or overweight patients is difficult to achieve, and studies investigating fluid management strategies (such as goal-directed fluid therapy) should assess the effects of BMI on outcomes^28^. Intensive postoperative care, early mobilization, physiotherapy and novel methods to reduce SSI, above and beyond laparoscopy, should be investigated. Previous research has associated alterations in tissue oxygen tension, the pharmacokinetic distribution of prophylactic antibiotics and impaired immunological response in obese patients with a heightened risk of SSI^29–31^. There are considerable additional costs associated with SSI, adding considerable length and costs to inpatient stay^32^. Education, novel wound devices and antibiotic delivery methods may be beneficial in reducing SSI in obese patients with cancer.

## Collaborators

Writing and Steering Group (asterisk indicates joint first author): T. M. Drake*, D. Nepogodiev*, S. J. Chapman*, J. C. Glasbey*, C. Khatri*, C. Y. Kong*, H. A. Claireaux, M. F. Bath, M. Mohan, L. McNamee, M. Kelly, H. Mitchell, J. E. Fitzgerald, E. M. Harrison, A. Bhangu (overall guarantor).

Statistical analysis: T. M. Drake, A. Bhangu, E. M. Harrison (statistical guarantor).

Local leads: H. A. Claireaux (University of Bristol, Bristol); I. Antoniou (Hull York Medical School, Hull); R. Dean (Leicester Medical School, Leicester); N. Davies (University of East Anglia, Norwich); S. Trecarten, I. Henderson (University of Nottingham, Nottingham); C. Holmes (University of Sheffield, Sheffield); J. Wylie, R. H. Shuttleworth (Queen's University Belfast, Belfast); A. Jindal (University of Limerick, Limerick); F. Hughes, P. Gouda (National University of Ireland, Galway); L. McNamee (Royal College of Surgeons in Ireland, Dublin); R. Fleck (Trinity College, Dublin); M. Hanrahan (University College Cork, Cork); P. Karunakaran (University College Dublin, Dublin); J. H. Chen, M. C. Sykes (Imperial College, London); R. K. Sethi, S. Suresh (King's College, London); P. Patel, M. Patel (Queen Mary University, London); R. K. Varma, J. Mushtaq (St George's University of London, London); B. Gundogan (University College London, London); W. Bolton (University of Leeds, Leeds); M. Mohan, T. Khan (University of Liverpool, Liverpool); J. Burke, R. Morley (University of Manchester, Manchester); N. Favero (Newcastle University Medical School, Newcastle upon Tyne); R. Adams (University of Aberdeen, Aberdeen); V. Thirumal (University of Dundee, Dundee); E. D. Kennedy (University of Edinburgh, Edinburgh); K. K. Ong, Y. H. Tan (University of Glasgow, Glasgow); J. Gabriel (Brighton and Sussex Medical School, Brighton); A. Bakhsh, J. Y. L. Low (Peninsula, Exeter and Plymouth); A. Yener (Southampton Medical School, Southampton); V. Paraoan (University of Cambridge, Cambridge); R. Preece, T. W. Tilston (Cardiff University, Cardiff); E. Cumber (University of Oxford, Oxford); S. Dean (Swansea University, Swansea); T. Ross, E. McCance (University of Birmingham, Birmingham); H. Amin (Keele University, Keele); L. Satterthwaite (University of Warwick, Coventry).

Other collaborators: K. D. Clement, R. Gratton, E. D. Mills, S. M. Chiu, G. Hung, N. M. Rafiq, J. D. B. Hayes, K. L. Robertson, K. Dynes (Aberdeen Royal Infirmary, Aberdeen); H. C. Huang, S. Assadullah, J. W. Duncumb, R. D. C. Moon, S. X. Poo, J. K. Mehta, K. R. Joshi, R. Callan, J. M. Norris, N. J. Chilvers, H. Keevil, P. Jull (Addenbrooke's Hospital, Cambridge); S. Mallick, D. Elf, L. Carr (Airedale General Hospital, Steeton); C. Player, E. C. Barton, A. L. Martin, S. G. Ratu, E. J. Roberts, P. N. Phan, A. R. Dyal, J. E. Rogers, A. D. Henson (Alexandra Hospital, Redditch); N. B. Reid, D. Burke, G. Culleton, S. Lynne, D. Burke (Antrim Area Hospital, Antrim); S. Mansoor, C. Brennan, R. Blessed, C. Holloway, A. Hill, T. Goldsmith, S. Mackin (Arrowe Park Hospital, Wirral); S. Kim, E. Woin, G. Brent, J. Coffin, O. Ziff (Barnet Hospital, Barnet); Z. Momoh, R. Debenham, M. Ahmed (Basingstoke and North Hampshire Hospital, Basingstoke); C. S. Yong, J. C. Wan, H. C. Copley, P. Raut, F. I. Chaudhry (Bedford Hospital, Bedford); R. H. Shuttleworth, G. Nixon, C. Dorman, R. Tan, S. Kanabar, N. Canning, M. Dolaghan, N. Bell, M. McMenamin (Belfast City Hospital, Belfast); A. Chhabra, K. Duke, L. Turner, T. Patel, L. S. Chew, M. Mirza, S. Lunawat, B. Oremule (Blackpool Victoria Hospital, Blackpool); N. Ward, M. Khan (Bolton Royal Hospital, Bolton); E. T. Tan, D. Maclennan, R. J. McGregor, E. G. Chisholm, E. J. Griffin, L. Bell (Borders General Hospital, Melrose); B. A. Hughes, J. Davies, H. Haq, H. Ahmed, N. Ungcharoen, C. Whacha, R. Thethi (Bradford Royal Infirmary, Bradford); R. M. Markham, A. H. Y. Lee, E. Batt, N. P. Bullock, C. T. Francescon, J. E. Davies, N. M. Shafiq (Bristol Royal Infirmary, Bristol); J. Zhao, S. Vivekanantham, I. Barai, J. L. Y. Allen, D. C. Marshall, C. J. McIntyre, H. C. P. Wilson, A. J. Ashton, C. Lek (Charing Cross Hospital, London); N. Behar, M. Davis-Hall, N. Seneviratne, S. Kim, L. Esteve, M. Sirakaya, S. Ali, S. Pope, J. S. Ahn, A. Craig-McQuaide (Chelsea and Westminster Hospital, London); W. A. Gatfield, S. Leong, A. M. Demetri, A. L. Kerr (Cheltenham General Hospital, Cheltenham); C. Rees, J. Loveday, S. Liu, M. Wijesekera, D. Maru, M. Attalla, N. Smith (Chester Hospital, Chester); D. Brown, P. Sritharan, A. Shah, V. Charavanamuttu, G. Heppenstall-Harris, K. Ng, T. Raghvani, N. Rajan, K. Hulley (Colchester Hospital, Colchester); N. Moody, M. Williams, A. Cotton (Conquest Hospital, Hastings); M. Sharifpour, K. N. Lwin, M. Bright, A. R. Chitnis, M. Abdelhadi, A. D. Semana (County Hospital, Stafford); F. Morgan, R. Reid, J. Dickson, L. Anderson, R. McMullan, J. Dickson, N. Ahern, A. Asmadi, L. B. Anderson (Craigavon Area Hospital, Craigavon); J. Lua Boon Xuan, L. Crozier, S. McAleer (Daisy Hill Hospital, Newry); D. M. Lees, A. A. Adebayo, M. Das, A. H. Amphlett (Derriford Hospital, Plymouth); A. Al-Robeye, A. Valli, J. Khangura, A. Winarski, A. Ali, J. Khangura (Dewsbury Hospital, Dewsbury); H. Woodward, C. Gouldthrope, M. Turner, K. Sasapu (Diana Princess of Wales Hospital, Grimsby); M. Tonkins, J. R. L. Wild, M. Robinson, J. Hardie, R. Heminway, R. Narramore, N. Ramjeeawon, A. Hibberd (Doncaster Royal Infirmary, Doncaster); F. Winslow, W. Ho, B. F. Chong, K. Lim, S. Ho (Dumfries and Galloway Royal Infirmary, Dumfries); J. A. Crewdson, S. Singagireson, N. Kalra, F. Koumpa, H. Jhala, W. C. Soon, M. Karia, M. G. Rasiah, D. Xylas (Ealing Hospital, Southall); H. Gilbert, M. Sundar-Singh, J. Wills (Eastbourne District General Hospital, Eastbourne); J. Mushtaq, S. Akhtar, S. Patel, L. Hu, C. Brathwaite-Shirley, H. Nayee, O. Amin, T. Rangan, E. J. H. Turner (East Surrey Hospital, Redhill); C. McCrann, R. Shepherd, N. Patel, J. Prest-Smith, E. Auyoung, A. Murtaza, A. Coates (Epsom Hospital, Epsom); O. Prys-Jones, M. King, S. Gaffney, C. J. Dewdney, I. Nehikhare, J. Lavery (Forth Valley Royal Hospital, Larbert); J. Bassett, K. Davies, K. Ahmad, A. Collins, M. Acres, C. Egerton, T. Khan (Furness General Hospital, Barrow-in-Furness); K. Cheng, X. Chen, N. Chan, A. Sheldon, S. Khan, J. Empey, E. Ingram, A. Malik, M. Johnstone (Gartnavel General Hospital, Glasgow); R. Goodier, J. P. Shah, J. E. Giles, J. A. Sanders, S. W. McLure, S. Pal, A. Rangedara, A. N. Baker, C. A. Asbjoernsen (George Eliot Hospital, Nuneaton); C. Girling, L. Gray, L. Gauntlett, C. Joyner, S. Qureshi, S. Dean (Glangwili General Hospital, Carmarthen); Y. P. Mogan, J. C. K. Ng, A. N. Kumar, J. H. Park, D. Tan, K. P. Choo, K. P. Raman, P. Buakuma, C. Xiao, S. Govinden (Glasgow Royal Infirmary, Glasgow); O. D. Thompson, M. A. Charalambos, E. Brown, R. B. Karsan, T. Dogra, L. M. Bullman, P. M. Dawson (Gloucestershire Royal Hospital, Gloucester); A. L. Frank, H. Abid, L. Tung, U. Qureshi, A. Tahmina, B. W. Matthews (Good Hope Hospital, Sutton Coldfield); R. T. Harris, A. O'Connor, K. Mazan, S. Iqbal, S. A. Stanger, J. D. Thompson (Great Western Hospital, Swindon); J. A. L. Sullivan, E. Uppal, A. MacAskill, F. A. Bamgbose, C. Neophytou, A. F. Carroll, C. W. Rookes, U. Datta, A. J. Dhutia (Hammersmith Hospital, Hammersmith); S. Rashid, N. Ahmed, T. Lo (Harrogate District Hospital, Harrogate); S. Bhanderi, C. D. Blore, S. Ahmed, H. Shaheen, S. Abburu, S. Majid, Z. Abbas, S. S. Talukdar, S. Ahmed (Heartlands Hospital, Birmingham); L. J. Burney, J. B. Patel, O. Al-Obaedi, A. W. Roberts, O. Al-Obaedi, S. Mahboob (Hereford County Hospital, Hereford); B. Singh, S. Sheth, P. Karia, A. Prabhudesai, K. Kow, K. Koysombat, S. Wang, P. Morrison, Y. Maheswaran, P. Keane (Hillingdon Hospital, Uxbridge); P. C. Copley, O. Brewster, G. X. Xu, P. Harries, C. Wall (Hinchingbrooke Hospital, Huntingdon); A. Al-Mousawi, S. Bonsu, P. Cunha, T. Ward, J. Paul, K. Nadanakumaran, S. Tayeh, T. Ward, H. Holyoak, J. Remedios, K. Theodoropoulou, T. Ward (Homerton University Hospital, London); A. Luhishi, L. Jacob, F. Long, A. Atayi, S. Sarwar, O. Parker (Huddersfield Royal Infirmary, Huddersfield); J. Harvey, H. Ross, R. Rampal, G. Thomas, P. Vanmali, C. McGowan, J. Stein (Hull Royal Infirmary, Hull, and Castle Hill Hospital, Cottingham); V. Robertson, L. Carthew, V. Teng, J. Fong (Inverclyde Royal Hospital, Greenock); A. N. Street, C. E. Thakker (Ipswich Hospital, Ipswich); D. O'Reilly, M. Bravo, A. Pizzolato, H. A. Khokhar, M. Ryan, L. Cheskes, R. Carr, A. E. Salih (James Connolly Hospital, Dublin); S. Bassiony, R. Yuen, D. Chrastek, H. Rosen O'Sullivan, A. Amajuoyi, A. Wang, O. Sitta, J. Wye (James Paget University Hospital, Great Yarmouth); M. A. Qamar, C. Major, A. Kaushal (Kent and Canterbury Hospital, Canterbury); C. Morgan, M. Petrarca, R. Allot, K. Verma, S. Dutt, R. Allot, C. P. Chilima, S. Peroos, R. Allot (King George Hospital, Ilford); S. R. Kosasih, H. Chin, L. Ashken, R. J. Pearse R. A. O'Loughlin, A. Menon, K. Singh, J. Norton (King's College Hospital, London); R. Sagar, N. Jathanna, L. Rothwell, N. Watson, F. Harding, P. Dube (King's Mill Hospital, Sutton-in-Ashfield); H. Khalid, N. Punjabi, M. Sagmeister, P. Gill, S. Shahid, S. Hudson-Phillips, D. George, J. Ashwood, T. Lewis (Kingston Hospital, Kingston upon Thames); M. Dhar, P. Sangal, I. A. Rhema, D. Kotecha, R. Dean, Z. Afzal, J. A. Syeed, E. Prakash, P. Jalota, R. Dean (Leicester Royal Infirmary, Leicester); J. Herron, L. Kimani, A. Delport, A. Shukla (Lincoln County Hospital, Lincoln); V. Agarwal, S. Parthiban, H. Thakur, W. Cymes, S. Rinkoff (Lister Hospital, Stevenage); J. A. Turnbull, M. Hayat, S. Darr, U. Khan, J. Lim, A. Higgins (Manchester Royal Infirmary, Manchester); G. Lakshmipathy, B. Forte, E. Canning, A. Jaitley, J. Lamont, E. Toner, A. Ghaffar, M. McDowell, D. Salmon (Mater Infirmorum Hospital, Belfast); P. Gouda, O. O'Carroll, A. Khan, M. E. Kelly, K. Clesham, C. Palmer, R. Lyons, M. E. Kelly, A. Bell, R. Chin, R. M. Waldron, M. E. Kelly (Mayo General Hospital, Castlebar); A. Trimble, S. E. Cox, U. Ashfaq, J. Campbell, R. B. S. Holliday, G. McCabe (Monklands Hospital, Airdrie); F. Morris, R. Priestland, S. Dean, O. K. Vernon, A. Ledsam, R. Vaughan (Morriston Hospital, Swansea); D. Lim, Z. R. Bakewell, R. K. Hughes (Musgrove Park Hospital, Taunton); R. M. Koshy, H. R. Jackson, P. Narayan, A. E. Cardwell, C. L. Jubainville, T. Arif, L. E. Elliott, V. Gupta, T. Arif (New Cross Hospital, Wolverhampton); G. Bhaskaran, K. Singh, A. Odeleye, F. Ahmed, R. Shah, A. Odeleye, J. Pickard, Y. N. Suleman, A. Odeleye (Newham University Hospital, London); A. S. North, L. F. McClymont, N. Hussain, I. Ibrahim, G. S. Ng, V. Wong, A. E. Lim, L. N. Harris, T. Tharmachandirar, D. Mittapalli (Ninewells Hospital, Dundee); V. Patel, M. Lakhani (Nobles Hospital, Isle of Man); N. Davies, H. Z. Bazeer, V. Narwani, K. K. Sandhu, L. R. Wingfield, S. Gentry, H. Adjei, M. Bhatti, L. Braganza (Norfolk and Norwich University Hospital, Norwich); J. Barnes, S. Mistry, G. Chillarge, S. Stokes, J. Cleere, S. Wadanamby, A. M. Bucko, J. Meek, N. Boxall, E. G. Heywood, J. J. Wiltshire, C. Toh, A. E. Ward, B. N. Shurovi, T. M. Drake (Northern General Hospital, Sheffield); D. Horth, B. Y. Patel, B. Ali, T. Spencer, T. Axelson, L. Kretzmer, C. Chhina (North Manchester General Hospital, Manchester); C. Anandarajah, T. Fautz, C. Horst (North Middlesex University Hospital NHS Trust, Edmonton); A. A. Thevathasan, J. Q. Ng, F. Hirst (North Tyneside General Hospital, North Shields); C. F. Brewer, A. E. Logan, J. W. Lockey, P. R. Forrest, N. Keelty, A. D. Wood, L. R. Springford, P. Avery, T. M. Schulz, T. P. Bemand, L. Howells (Northwick Park Hospital, Harrow); H. Collier, A. Khajuria, R. G. Tharakan, S. Parsons (Nottingham City Hospital, Nottingham); A. M. Buchan, R. J. McGalliard, J. D. Mason, O. J. Cundy, N. Li, N. A. Redgrave, R. P. Watson, T. P. Pezas, Y. F. Dennis, E. Segall, M. Hameed, A. S. Lynch (John Radcliffe and Churchill Hospitals, Oxford); M. Chamberlain, F. S. Peck, Y. N. Neo, G. Russell, M. Elseedawy, S. Lee, N. L. Foster, Y. H. Soo, L. Puan (Perth Royal Infirmary, Perth); R. Dennis, H. Goradia, A. Qureshi (Peterborough City Hospital, Peterborough); S. Osman, T. Reeves, L. Dinsmore, M. Marsden, Q. Lu, T. Pitts-Tucker (Portsmouth Hospitals NHS Trust Queen Alexandra Hospital, Portsmouth); C. E. Dunn, R. A. Walford, E. Heathcote, R. Martin, A. Pericleous, K. Brzyska, K. G. Reid, M. R. Williams, N. Wetherall (Prince Charles Hospital, Merthyr Tydfil); E. McAleer, D. Thomas, R. Kiff, C. Gouldthrope (Princess of Wales Hospital, Bridgend); S. Milne, M. J. V. Holmes, S. Stokes, J. Bartlett, J. Lucas de Carvalho, T. Bloomfield (Princess Royal Hospital, Haywards Heath); F. Tongo, R. H. Bremner N. Yong, B. A. Atraszkiewicz, A. Mehdi, M. Tahir, G. X. J. Sherliker, A. K. Tear, A. Pandey (Princess Royal University Hospital, Bromley); A. Broyd, H. M. Omer, M. Raphael, W. W. Chaudhry, S. Shahidi, A. S. Jawad, C. K. Gill, I. Hindle Fisher, I. Adeleja, I. J. Clark, G. E. Aidoo-Micah (Queen Elizabeth Hospital, Birmingham); P. W. Stather, G. J. Salam, T. E. Glover, G. Deas, N. K. Sim, R. D. Obute, W. M. Wynell-Mayow (Queen Elizabeth Hospital, King's Lynn); M. S. Sait, N. Mitha, G. L. de Bernier, M. Siddiqui, R. Shaunak, A. Wali, G. Cuthbert (Queen Elizabeth Hospital, Woolwich); R. Bhudia, E. Webb, S. Shah, N. Ansari, M. Perera, N. Kelly (Queen's Hospital, Romford); R. McAllister, G. H. Stanley, C. P. Keane, V. Shatkar, C. Maxwell-Armstrong (Queen's Medical Centre, Nottingham); L. A. Henderson, N. Maple, R. Manson, R. D. Adams, E. Brown, E. Semple (Raigmore Hospital, Inverness); M. Mills, A. Daoub, A. Marsh, A. Ramnarine, J. Hartley, M. Malaj (Rotherham General Hospital, Rotherham); P. D. Jewell, E. A. Whatling, N. Hitchen, M. Chen (Royal Berkshire Hospital, Reading); B. Goh, J. Fern, S. Rogers, L. Derbyshire (Royal Blackburn Hospital, Blackburn); D. T. Robertson, N. Abuhussein, P. Deekonda, A. Abid, A. Bakhsh (Royal Devon and Exeter Hospital, Exeter); P. L. M. Harrison, L. Aildasani, H. Turley (Royal Glamorgan Hospital, Ynysmaerdy); M. A. Sherif, G. Pandey, J. J. Filby, A. Johnston, E. Burke, M. Mohamud, K. Gohil, A. Y. Tsui, R. Singh (Royal Gwent Hospital, Newport); S. J. Lim, K. O'Sullivan, L. L. McKelvey, S. O'Neill, H. F. Roberts, F. S. Brown, Y. Cao, R. T. Buckle, Y. Liew, S. Sii, C. M. Ventre, C. J. Graham, T. Filipescu (Royal Infirmary of Edinburgh, Edinburgh); A. Yousif, R. Dawar, A. Wright, M. Peters, R. Varley, S. Owczarek, S. Hartley (Royal Lancaster Infirmary, Lancaster); M. Khattak, A. Iqbal, M. Ali, B. Durrani, Y. Narang, G. S. Bethell, L. Horne, R. Pinto (Royal Liverpool Hospital, Liverpool); K. Nicholls, I. Kisyov, H. D. Torrance, P. Patel, M. Patel, W. English, S. M. Lakhani, S. F. Ashraf, M. Venn (Royal London Hospital, London); V. Elangovan, Z. Kazmi, J. Brecher, S. Sukumar, A. Mastan, A. Mortimer, J. Parker, J. Boyle (Royal Preston Hospital, Preston); M. Elkawafi, J. Beckett, A. Mohite, A. Narain, E. Mazumdar, A. Sreh, A. Hague, D. Weinberg L. Fletcher (Royal Shrewsbury Hospital, Shrewsbury); M. Steel, H. Shufflebotham, M. Masood, Y. Sinha, H. Amin, C. Jenvey, H. Kitt, R. Slade, A. R. Craig, C. Deall, Y. Sinha (Royal Stoke University Hospital, Stoke-on-Trent); J. Gabriel, T. Reakes, J. Chervenkoff, E. Strange, M. O'Bryan, C. Murkin, D. Joshi, E. Strange, T. Bergara, S. Naqib, D. Wylam, E. Strange (Royal Sussex County Hospital, Brighton); S. E. Scotcher, C. M. Hewitt, M. T. Stoddart, A. Kerai, A. J. Trist, S. J. Cole, C. L. Knight, S. Stevens, G. E. Cooper (Royal United Hospital, Bath); R. Ingham, J. Dobson, J. Wylie, A. O'Kane, J. Moradzadeh, A. Duffy, C. Henderson, S. Ashraf, C. McLaughin (Royal Victoria Hospital, Belfast); T. C. Hoskins, R. S. Reehal, L. R. Bookless, R. C. McLean, E. J. Stone (Royal Victoria Infirmary, Newcastle upon Tyne); E. V. Wright, H. R. Abdikadir, C. Roberts, O. Spence, M. Srikantharajah, M. Patel, E. M. Ruiz, J. H. Matthews, E. Gardner, C. Roberts (Russell's Hall Hospital, Dudley); E. Hester, P. Naran, R. Simpson, M. Minhas, E. Cornish, S. A. Semnani, D. Rojoa (Salford Royal Hospital, Salford); A. Radotra, J. Eraifej, K. Eparh, D. N. E. Smith, B. D. Mistry, S. L. Hickling, A. Bhangu (Sandwell General Hospital, West Bromwich); W. Din, C. Liu, P. Mithrakumar, V. Mirdavoudi (Scarborough Hospital, Scarborough); M. Rashid, C. Mcgenity, O. Hussain, M. Kadicheeni, H. Gardner, N. Anim-Addo, J. Pearce, A. Aslanyan, C. Ntala, T. Sorah, J. Parkin, M. Alizadeh (Scunthorpe Hospital, Scunthorpe); A. White, F. Edozie, J. Johnston, A. Kahar, V. Navayogaarajah, B. Patel, D. Carter, P. Khonsari, A. Burgess, B. Patel (Southend Hospital, Westcliff-on-Sea); C. Kong, A. Ponweera, A. Cody, Y. Tan, A. Y. L. Ng, A. Croall, C. Allan, S. Ng, V. Raghuvir (Southern General Hospital, Glasgow); R. Telfer, A. D. Greenhalgh, C. N. McKerr, M. A. Edison, B. A. Patel, K. Dear, M. R. Hardy (Southmead Hospital, Bristol); P. Williams, S. Hassan, U. Sajjad (Southport Hospital, Southport); E. M. O'Neill, S. Lopes, L. Healy (South Tipperary Hospital, Tipperary); N. Jamal, S. Tan, D. Lazenby, S. B. Husnoo, S. Beecroft, T. Sarvanandan (Stepping Hill Hospital, Stockport); C. Weston, N. Bassam, S. Rabinthiran, U. Hayat, L. Ng, D. Varma (St George's Hospital, London); M. Sukkari, A. Mian, A. Coates, A. Omar, J. W. Kim, A. Coates, J. Sellathurai, J. Mahmood (St Helier Hospital, Carshalton); C. O'Connell, R. Bose, H. Heneghan, P. Lalor, J. Matheson, C. Doherty, C. Cullen, D. Cooper, S. Angelov, C. Drislane (St James' Hospital, Dublin); A. C. D. Smith, A. Kreibich, E. Palkhi, A. Durr, A. Lotfallah, D. Gold, E. Mckean, A. Durr, A. Dhanji, A. Anilkumar, A. Thacoor, A. Durr (St James's University Hospital, Leeds); Z. H. Siddiqui, S. Lim, A. Piquet, S. M. Anderson, A. Jindal, D. R. McCormack, J. Gulati, A. Ibrahim, A. Jindal, S. E. Murray, S. L. Walsh, A. McGrath (St Luke's Hospital, Limerick); P. Ziprin, E. Y. Chua, C. N. Lou, J. Bloomer, H. R. Paine, D. Osei-Kuffour, C. J. White, A. Szczap, S. Gokani, K. Patel (St Mary's Hospital, London); M. K. Malys, A. Reed, G. E. Torlot, E. M. Cumber (Stoke Mandeville Hospital, Aylesbury, and High Wycombe Hospital, High Wycombe); A. Charania, S. Ahmad, N. Varma, H. Cheema, L. Austreng, H. Petra, M. Chaudhary (St Peter's Hospital, Chertsey); M. I. Zegeye, F. Cheung, D. Coffey, R. S. Heer, S. Singh, E. Seager, S. Cumming, R. S. Suresh, S. Verma, I. B. Ptacek, A. M. Gwozdz (St Thomas' Hospital, London); T. Yang, A. A. Khetarpal, S. Shumon, T. M. P. Fung, W. Leung, P. Kwang, L. Chew, W. Loke, A. Curran (Sunderland Royal Hospital, Sunderland); C. Chan (Tameside General Hospital, Ashton-under-Lyne); C. McGarrigle, K. Mohan, S. Cullen, E. Wong, C. Toale, D. Collins, N. Keane, B. P. Traynor, D. Shanahan (The Adelaide and Meath Hospital, Dublin); A. Yan, D. J. Jafree, C. Topham S. Mitrasinovic, S. Omara, B. Gundogan, G. Bingham, P. M. Lykoudis, B. H. Miranda (The Royal Free Hospital, London); K. Whitehurst, G. Kumaran, Y. Devabalan, H. Aziz, M. Shoa, S. Dindyal (The Whittington Hospital, London); J. A. Yates, I. Bernstein, G. Rattan, J. A. Yates (Tullamore Hospital, Tullamore); R. Coulson, S. Stezaker, A. Isaac, M. Salem, A. McBride, A. Isaac, H. McFarlane, L. Yow, J. MacDonald (Ulster Hospital, Belfast); R. D. Bartlett, S. Turaga, U. White, W. Liew, N. Yim (University College London Hospital, London); A. Ang, A. Simpson, D. McAuley, E. Craig, L. Murphy, P. Shepherd, J. Y. Kee, A. Abdulmajid, A. Chung (University Hospital Ayr, Ayr); H. L. Warwick, A. Livesey, P. Holton, M. D. Theodoreson, S. L. Jenkin, J. Turner, J. H. Entwisle, S. T. Marchal, S. O'Connor, H. K. Blege (University Hospital Coventry and Warwickshire, Coventry); J. M. Aithie, L. M. Sabine, G. E. Stewart, S. Jackson (University Hospital Crosshouse, Crosshouse); A. Kishore, C. M. Lankage, F. Acquaah, H. L. Joyce (University Hospital Lewisham, Lewisham); A. Jindal, K. L. McKevitt, C. J. Coffey, A. S. Fawaz, K. S. Dolbec, D. A. O'Sullivan, J. M. Geraghty (University Hospital Limerick, Limerick); E. Lim, L. Bolton, D. FitzPatrick, C. Robinson, T. Ramtoola, S. Collinson (University Hospital of South Manchester, Manchester); L. Grundy, P. M. McEnhill, G. S. Harbhajan Singh, D. Loughran, D. M. Golding, R. E. Keeling, R. P. Williams, R. D. J. Whitham, S. Yoganathan, R. Nachiappan, R. J. Egan (University Hospital of Wales, Cardiff); R. Owasil, M. L. Kwan, A. He, R. W. Goh, R. Bhome, H. Wilson, P. J. Teoh, K. Raji, T. Reeves, N. Jayakody, J. Matthams (University Hospital Southampton NHS Foundation Trust, Southampton); J. Chong, S. Tan, C. Y. Luk, R. J. Greig, M. Trail, G. Charalambous, V. Thirumal, A. S. Rocke, N. Gardiner (Victoria Hospital, Kirkcaldy); F. Bulley, N. Warren, E. Brennan, P. Fergurson, R. Wilson, H. Whittingham (Victoria Infirmary, Glasgow); E. J. Brown, R. Khanijau, K. Gandhi, S. Morris, A. J. Boulton, N. Chandan, A. E. Barthorpe, R. Maamari, S. Sandhu (Walsall Manor Hospital, Walsall); M. McCann, L. Higgs, V. Balian, C. Reeder, C. Diaper, V. Balian, T. Sale, H. Ali, V. Balian (Warrington Hospital, Warrington); C. H. Archer, A. K. Clarke, J. Heskin, P. C. Hurst, J. D. Farmer, L. D. O'Flynn, L. Doan, B. A. Shuker, G. D. Stott (Warwick Hospital, Warwick); N. A. Vithanage, K. A. Hoban, P. N. Nesargikar, H. R. Kennedy, C. M. Grossart, E. S. M. Tan, C. S. D. Roy, P. Sim, K. E. Leslie (Western General Hospital, Edinburgh); D. Sim, M. H. Abul, N. Cody, A. Y. Tay, E. Woon, S. Sng, J. Mah, J. Robson (Western Infirmary, Glasgow); E. Shakweh, V. C. Wing, H. Mills, M. M. Li, T. R. Barrow, S. Balaji, H. E. M. Jordan, C. Phillips, H. Naveed (West Middlesex Hospital, Isleworth); S. Hirani, A. Tai, R. Ratnakumaran, A. Sahathevan, A. M. A. Shafi, M. Seedat, R. Weaver (Whipps Cross Hospital, London); A. Batho, R. Punj, H. Selvachandran, N. Bhatt, S. Botchey, Z. Khonat, K. Brennan (Whiston Hospital, Prescot); K. K. Ong, C. J. Morrison, E. Devlin, A. Linton, E. Galloway, S. McGarvie, N. Ramsay (Wishaw General Hospital, Wishaw); H. D. McRobbie, H. Whewell, W. Dean (Wrexham Maelor Hospital, Wrexham); S. Nelaj, M. Eragat, A. Mishra, T. Kane, M. Zuhair, M. Wells, D. Wilkinson, N. Woodcock (York Hospital, York); E. Sun, N. Aziz, M. K. Abd Ghaffar (Ysbyty Gwynedd, Bangor).

## Supplementary Material

bjs10203-sup-0001-AppendixS1 **Table S1** Derivation of the operative risk class by mortality rate
**Table S2** Comparison of normal-weight, overweight and obese patients undergoing surgery for malignancy
**Table S3** Comparison across World Health Organization obesity subgroups I, II and III
**Table S4** Thirty-day mortality by operative risk class across body mass index groups

## Figures and Tables

**Figure:**