# Supplementary material for: Multicentre prospective cohort study of body mass index and postoperative complications following gastrointestinal surgery
Source: Br J Surg. 2016 Jun 20;103(9):1157–72. doi: 10.1002/bjs.10203 (PMC4973675; doi:10.1002/bjs.10203)
Supplement: bjs10203-sup-0001-AppendixS1 — Table S1 Derivation of the operative risk class by mortality rate Table S2 Comparison of normal-weight, overweight and obese patients undergoing surgery for malignancy Table S3 Comparison across World Health Organization obesity subgroups I, II and III Table S4 Thirty-day mortality by operative risk class across body mass index groups [file bjs10203-sup-0001-appendixs1.docx]

**BJS10203**

**Multicentre prospective cohort study on body mass index and postoperative complications following gastrointestinal surgery**

STARSurg Collaborative

**Table S1** Derivation of the operative risk class by mortality rate

| OPCS-4 code | Description | Operative mortality (%) | Mortality rank (low to high) | Operative risk class |
| --- | --- | --- | --- | --- |
| J07 | Other open operations on liver | 0.0 | 1 | Low |
| G30 | Plastic operations on stomach | 0.1 | 2 | Low |
| G40 | Incision of pylorus | 0.2 | 3 | Low |
| H01 | Emergency excision of appendix | 0.2 | 4 | Low |
| T20 | Primary repair of inguinal hernia | 0.2 | 5 | Low |
| G24 | Antireflux operations | 0.3 | 6 | Low |
| J18 | Excision of gallbladder | 0.3 | 7 | Low |
| H35 | Fixation of rectum for prolapse | 0.8 | 8 | Low |
| H46 | Other operations on rectum | 0.8 | 9 | Low |
| G75 | Attention to artificial opening into ileum | 1.6 | 10 | Moderate |
| T55 | Release of fascia | 1.8 | 11 | Moderate |
| X14 | Clearance of pelvis | 1.9 | 12 | Moderate |
| G23 | Repair of diaphragmatic hernia | 2.0 | 13 | Moderate |
| H04 | Total excision of colon and rectum | 2.0 | 14 | Moderate |
| H12 | Extirpation of lesion of colon | 2.0 | 15 | Moderate |
| J02 | Partial excision of liver | 2.4 | 16 | Moderate |
| G21 | Other operations on oesophagus | 2.6 | 17 | Moderate |
| J55 | Total excision of pancreas | 3.3 | 18 | Moderate |
| J56 | Excision of head of pancreas | 4.1 | 19 | Moderate |
| J12 | Other therapeutic percutaneous operations on liver | 5.0 | 20 | Moderate |
| G01 | Excision of oesophagus and stomach | 5.1 | 21 | Moderate |
| H10 | Excision of sigmoid colon | 5.3 | 22 | Moderate |
| G03 | Partial excision of oesophagus | 5.5 | 23 | Moderate |
| H15 | Other exteriorization of colon | 5.5 | 24 | Moderate |
| G51 | Bypass of duodenum | 5.6 | 25 | Moderate |
| H33 | Excision of rectum | 5.6 | 26 | Moderate |
| J16 | Other operations on liver | 5.9 | 27 | Moderate |
| H09 | Excision of left hemicolon | 6.1 | 28 | Moderate |
| J30 | Connection of common bile duct | 6.1 | 29 | Moderate |
| G34 | Artificial opening into stomach | 6.6 | 30 | Moderate |
| J61 | Open drainage of lesion of pancreas | 7.0 | 31 | Moderate |
| G28 | Open extirpation of lesion of stomach | 7.2 | 32 | Moderate |
| G27 | Total excision of stomach | 7.3 | 33 | Moderate |
| J33 | Incision of bile duct | 7.7 | 34 | Moderate |
| H19 | Other open operations on colon | 9.5 | 35 | Moderate |
| G60 | Artificial opening into jejunum | 10.0 | 36 | High |
| H06 | Extended excision of right hemicolon | 10.0 | 37 | High |
| G63 | Other open operations on jejunum | 10.1 | 38 | High |
| G67 | Other operations on jejunum | 10.1 | 39 | High |
| J24 | Therapeutic percutaneous operations on gallbladder | 10.5 | 40 | High |
| G58 | Excision of jejunum | 11.0 | 41 | High |
| G74 | Creation of artificial opening into ileum | 11.7 | 42 | High |
| G69 | Excision of ileum | 12.2 | 43 | High |
| G49 | Excision of duodenum | 12.3 | 44 | High |
| H08 | Excision of transverse colon | 12.6 | 45 | High |
| H29 | Subtotal excision of colon | 13.3 | 46 | High |
| G35 | Operations on ulcer of stomach | 15.6 | 47 | High |
| T30 | Opening of abdomen | 19.9 | 48 | High |
| H13 | Bypass of colon | 24.8 | 49 | High |

**Table S2** Comparison of normal-weight, overweight and obese patients undergoing surgery for malignancy

|  | Normal  (*n* = 742) | Overweight  (*n* = 830) | Obese  (*n* = 557) | *P*† |
| --- | --- | --- | --- | --- |
| Age (years)* | 69.3(36.5) | 67.0(11.4) | 65.4(11.5) | < 0.001† |
| Sex |  |  |  | < 0.001 |
| M | 398 (53.6) | 538 (64.8) | 334 (60.0) |  |
| F | 344 (46.4) | 292 (35.2) | 223 (40.0) |  |
| ASA fitness grade |  |  |  | 0.001 |
| I | 94 (12.7) | 119 (14.3) | 44 (7.9) |  |
| II | 401 (54.0) | 470 (56.6) | 298 (53.5) |  |
| III | 212 (28.6) | 217 (26.1) | 196 (35.2) |  |
| IV | 25 (3.4) | 17 (2.0) | 17 (3.1) |  |
| V | 4 (0.5) | 1 (0.1) | 0 (0) |  |
| Missing | 6 (0.8) | 6 (0.7) | 2 (0.4) |  |
| Smoking status |  |  |  |  |
| Non-smoker | 607 (81.8) | 730 (88.0) | 490 (88.0) | 0.001 |
| Current smoker | 135 (18.2) | 100 (12.0) | 67 (12.0) |  |
| Revised Cardiac Risk Index |  |  |  |  |
| 0 | 542 (73.0) | 550 (66.3) | 318 (57.1) | < 0.001 |
| I | 155 (20.9) | 202 (24.3) | 178 (32.0) |  |
| ≥ II | 45 (6.1) | 78 (9.4) | 61 (11.0) |  |
| Operative risk class |  |  |  | 0.050 |
| Low | 16 (2.2) | 21 (2.5) | 26 (4.7) |  |
| Moderate | 476 (64.2) | 553 (66.6) | 349 (62.7) |  |
| High | 250 (33.7) | 256 (30.8) | 182 (32.7) |  |
| Urgency of surgery |  |  |  | 0.004 |
| Elective | 645 (86.9) | 737 (88.8) | 516 (92.6) |  |
| Emergency | 97 (13.1) | 93 (11.2) | 41 (7.4) |  |
| Operative approach |  |  |  | 0.025 |
| Open | 418 (56.3) | 416 (50.1) | 287 (51.5) |  |
| Open, laparoscopy-assisted | 45 (6.1) | 57 (6.9) | 34 (6.1) |  |
| Laparoscopy | 234 (31.5) | 305 (36.7) | 179 (32.1) |  |
| Laparoscopy converted to open | 43 (5.8) | 51 (6.1) | 56 (10.1) |  |
| Missing | 2 (0.3) | 1 (0.1) | 1 (0.2) |  |

Values in parentheses are percentages unless indicated otherwise; *values are mean(s.d.). ASA, American Society of Anesthesiologists. †χ^2^ test, except ‡Kruskal–Wallis test*.*

**Table S3** Comparison across World Health Organization obesity subgroups I, II and III

|  | Obese I  (*n* = 1484) | Obese II  (*n* = 647) | Obese III  (*n* = 616) | *P*† |
| --- | --- | --- | --- | --- |
| Age (years)* | 56.1(16.6) | 52.4(16.3) | 47.4(13.3) | < 0.001‡ |
| Sex |  |  |  | < 0.001 |
| M | 672 (45.3) | 202 (31.2) | 161 (26.1) |  |
| F | 812 (54.7) | 445 (68.8) | 455 (73.9) |  |
| ASA fitness grade |  |  |  | < 0.001 |
| I | 356 (24.0) | 126 (19.5) | 67 (10.9) |  |
| II | 765 (51.5) | 349 (53.9) | 327 (53.1) |  |
| III | 320 (21.6) | 153 (23.6) | 208 (33.8) |  |
| IV | 35 (2.4) | 14 (2.2) | 8 (1.3) |  |
| V | 1 (0.1) | 1 (0.2) | 3 (0.5) |  |
| Missing | 7 (0.5) | 4 (0.6) | 3 (0.5) |  |
| Smoking status |  |  |  | 0.269 |
| Non-smoker | 1241 (83.6) | 542 (83.8) | 532 (86.4) |  |
| Current smoker | 243 (16.4) | 105 (16.2) | 84 (13.6) |  |
| Revised Cardiac Risk Index |  |  |  | 0.240 |
| 0 | 1067 (71.9) | 453 (70.0) | 422 (68.5) |  |
| I | 317 (21.4) | 137 (21.2) | 153 (24.8) |  |
| ≥ II | 99 (6.7) | 57 (8.8) | 40 (6.5) |  |
| Missing | 1 (0.1) | 0 (0) | 1 (0.2) |  |
| Operative risk class |  |  |  | < 0.001 |
| Low | 709 (47.8) | 357 (55.2) | 424 (68.8) |  |
| Moderate | 420 (28.3) | 142 (21.9) | 113 (18.3) |  |
| High | 355 (23.9) | 148 (22.9) | 79 (12.8) |  |
| Diagnosis |  |  |  | < 0.001 |
| Benign | 1091 (73.5) | 541 (83.6) | 558 (90.6) |  |
| Malignant | 393 (26.5) | 106 (16.4) | 58 (9.4) |  |
| Urgency of surgery |  |  |  | < 0.001 |
| Elective | 952 (64.2) | 429 (66.3) | 520 (84.4) |  |
| Emergency | 532 (35.8) | 218 (33.7) | 96 (15.6) |  |
| Operative approach |  |  |  | < 0.001 |
| Open | 544 (36.7) | 191 (29.5) | 99 (16.1) |  |
| Laparoscopic | 938 (63.2) | 456 (70.5) | 517 (83.9) |  |
| Missing | 2 (0.1) | 0 (0) | 0 (0) |  |
| Major complications (Clavien Dindo III–V) |  |  |  | 0.269 |
| No | 1321 (89.0) | 590 (91.2) | 557 (90.4) |  |
| Yes | 163 (11.0) | 57 (8.8) | 59 (9.6) |  |
| Surgical-site infection |  |  |  | 0.141 |
| No | 1383 (93.2) | 605 (93.5) | 588 (95.5) |  |
| Yes | 101 (6.8) | 42 (6.5) | 28 (4.5) |  |

Values in parentheses are percentages unless indicated otherwise; *values are mean(s.d.). ASA, American Society of Anesthesiologists. †χ^2^ test, except ‡Kruskal–Wallis test*.*

**Table S4** Thirty-day mortality by operative risk class across body mass index groups

|  | Operative risk class | | |  |  |
| --- | --- | --- | --- | --- | --- |
|  | Low risk | Moderate risk | High risk | Total | *P** |
| All patients |  |  |  |  | < 0.001 |
| Alive | 3548 (99.8) | 2358 (98.0) | 1943 (97.0) | 7849 (98.5) |  |
| Dead | 8 (0.2) | 48 (2.0) | 60 (3.0) | 116 (1.5) |  |
| Normal weight |  |  |  |  | < 0.001 |
| Alive | 965 (99.9) | 832 (98.1) | 700 (95.8) | 2497 (98.1) |  |
| Dead | 1 (0.1) | 16 (1.9) | 31 (4.2) | 48 (1.9) |  |
| Overweight |  |  |  |  | < 0.001 |
| Alive | 1096 (99.6) | 860 (97.4) | 670 (97.1) | 2626 (98.2) |  |
| Dead | 4 (0.4) | 23 (2.6) | 20 (2.9) | 47 (1.8) |  |
| Obese |  |  |  |  | 0.001 |
| Alive | 1487 (99.8) | 666 (98.7) | 573 (98.5) | 2726 (99.2) |  |
| Dead | 3 (0.2) | 9 (1.3) | 9 (1.5) | 21 (0.8) |  |

Values in parentheses are percentages. *χ^2^ test.
